# Biomass and lipid production by the native green microalgae *Chlorella sorokiniana* in response to nutrients, light intensity, and carbon dioxide: experimental and modeling approach

**DOI:** 10.3389/fbioe.2023.1149762

**Published:** 2023-05-17

**Authors:** Carolina Montoya-Vallejo, Fernando León Guzmán Duque, Juan Carlos Quintero Díaz

**Affiliations:** Grupo de Bioprocesos, Departamento de Ingeniería Química, Universidad de Antioquia (UdeA), Medellín, Colombia

**Keywords:** *Chlorella sorokiniana*, lipids, mathematical model, microalgae, culture

## Abstract

**Introduction:** Microalgae are photosynthetic cells that can produce third-generation biofuels and other commercial compounds. Microalgal growth is influenced by two main parameters: light intensity and carbon dioxide concentration, which represent the energy and carbon source, respectively. For photosynthesis, the optimum values of abiotic factors vary among species.

**Methods:** In this study, the microalga *Chlorella sorokiniana* was isolated from a freshwater lake. It was identified using molecular analysis of the ribosomal internal transcribed spacer. A single-factor design of experiments in 250-mL Erlenmeyer flasks was used to evaluate which concentrations of nitrogen and phosphorus increase the production of biomass and lipids. The response surface methodology was used with a 3^2^-factorial design (light intensity and CO_2_ were used to evaluate its effect on biomass, lipid production, and specific growth rates, in 200-mL tubular photobioreactors (PBRs)).

**Results and Discussion:** Low levels of light lead to lipid accumulation, while higher levels of light lead to the synthesis of cell biomass. The highest biomass and lipid production were 0.705 ± 0.04 g/L and 55.1% ± 4.1%, respectively. A mathematical model was proposed in order to describe the main phenomena occurring in the culture, such as oxygen and CO_2_ mass transfer and the effect of light and nutrients on the growth of microalgae. The main novelties of this work were molecular identification of the strain, optimization of culture conditions for the indigenous microalgae species that were isolated, and formulation of a model that describes the behavior of the culture.

## 1 Introduction

The rapid increase in the world’s population has created immense pressure on the availability of sustainable energy resources. Achieving a balance between population growth and finite resources on Earth is currently one of the biggest challenges worldwide. Over the next 30 years, various issues such as energy shortages, the greenhouse effect, and climate change are expected to have a more severe impact on society. However, these challenges can be addressed by adopting sustainable and eco-friendly ways to generate energy. Microalgae hold significant promise in this respect (Khoo et al., 2021; Mat Aron et al., 2022). Microalgae are simple-structured photosynthetic organisms that can produce third-generation biofuels and other commercial compounds (cosmetics, pigments, ω-3 fatty acids, proteins, etc.). They also can be used as cathodic organisms in microbial fuel cells and produce hydrogen (Khoo et al., 2021; Wang et al., 2021). The advantages of microalgae include the following: i) the capacity to grow in seawater, brackish waters, and wastewaters using carbon dioxide as a carbon source, thereby mitigating CO_2_ emissions (Huang and Tan, 2014); removing wastewater pollutants; and generating value-added products; ii) resistance to a broad range of environmental conditions; iii) the capacity to be grown on land that is not appropriate for agriculture; and iv) high biomass productivity per unit area, high photosynthetic efficiency, and fast growth (Olguín, 2012; Abu-Ghosh et al., 2014; Collet et al., 2014; Ramos-Suárez et al., 2014; Park et al., 2015).

The number of lipids that are synthesized by microalgae depends on the inherent capacity of the species (genotype/phenotype) and on abiotic factors such as wavelength, light intensity, and composition of the culture medium (mainly carbon source, nitrogen, and phosphorus) (Metsoviti et al., 2020). Light (including its quality, intensity, and light/dark cycle) plays a critical role in affecting the growth and composition of microalgae biomass, particularly in regard to fatty acid and pigment profiles (Chia et al., 2018). Different research has identified that the light intensity required for proper algal growth is in the range of 40–1,240 μE/m^2^-s (Almomani, 2020). However, this could seem contradictory in terms of the lipid content because the response is strain-specific and will depend on other culture conditions. Biomass densities have been shown to increase when cultures of *Chlorella* sp. *and Nannochloropsis* sp. are exposed to light intensities above 117 μE/m^2^-s (de Jesus and Maciel Filho, 2017), as adequate lighting is crucial for the growth of microalgae. On the other hand, increasing light intensity could cause a decrease in microalgal growth. For example, increasing the light intensity above 150 μE/m^2^-s has resulted in a 10–25% and 20% decrease in growth rate and carbon uptake rate, respectively, because damage to light receptors lowers the efficiency of capturing solar light (Almomani, 2020). A clear positive relationship is not observed between light supply and the synthesis of carbohydrates or lipids in microalgae, although it has been established that irradiation can stimulate the synthesis of precursors for the synthesis of starch and sucrose. (de Jesus and Maciel Filho, 2017). Low light intensities are more favorable for lipid accumulation (de Jesus and Maciel Filho, 2017).

Nutrients are also very important for biomass growth and composition (Gouveia et al., 2014; Lee et al., 2015). Carbon is the main component of biomass (50% w/w approx.), and the supply of CO_2_ is important in the autotrophic cultivation of microalgae. The optimal values for carbon dioxide concentration vary not only among different microalgae strains but also for the same strain when grown under slightly different conditions (Aguirre and Bassi, 2013). Increasing CO_2_ levels increases algal growth in *Chlorella vulgaris* (de Jesus and Maciel Filho, 2017). However, if the CO_2_ supply exceeds the metabolic rate of the microalgae, the pH of the culture will decrease and become acidic, negatively impacting the growth of microalgae (Zhu and Huang, 2017). The optimal concentrations of CO_2_ for microalgae growth are around 10%, while higher concentrations (20%) could favor lipid accumulation. Furthermore, the lipid content of microalgae cultured under various levels of CO_2_ feeding is most likely influenced by differences in the microalgal species (Thawechai et al., 2016). The effects of N and P on biomass growth, lipid production, and CO_2_ uptake rate have been extensively studied experimentally, both of each single variable and also considering that the C:N:P ratio is an important factor in microalgae cultures (Almomani, 2020). Nitrogen and phosphate starvation is the most widely used strategy to improve lipid accumulation (Singh et al., 2016). In many microalgae, an increase in the carbon-to-nitrogen ratio results in lipid accumulation (Tongprawhan et al., 2014).

Microalgal growth can be modeled to describe the fate of the culture and depends on factors such as nutrient concentration, light radiation, and pH. The specific growth rate as a function of the incident light follows the Monod model, or it follows inhibition models when microalgae are exposed to high levels of light. The Monod model can also describe the effect of nutrients on growth. Regarding the gas–liquid transfer, the CO_2_ balance from the gas phase to cell biomass must be considered because CO_2_ is the sole carbon source in autotrophic cultures (Camacho Rubio et al., 1999; Yun and Park, 2003). Light and temperature are closely related, especially in outdoor cultures, and pH and CO_2_ have an important influence on microalgal growth and composition. While it would seem sensible to use multiparametric models, these are difficult to adjust because of the lack of data required for equal degrees of freedom (Darvehei et al., 2018).

In this study, autotrophic culture experiments using an isolated wild strain of *Chlorella sorokiniana* were conducted in order to investigate the effects of different nitrogen/phosphorus and carbon dioxide concentrations and different light intensities on growth and lipid production, using the response surface methodology (RSM). Additionally, phenomenological-based models for *C. sorokiniana* were constructed to study the lipid and biomass production of the microalgae.

## 2 Materials and methods

### 2.1 Isolation and identification of the microalgal strain

The microalgae culture of *C. sorokiniana* was isolated from a freshwater lake located in Medellín (Colombia). The isolated strain was made axenic by continuous sub-culturing using a modified Chu 13 medium (Yeesang and Cheirsilp, 2011) on agar plates supplemented with ampicillin and kanamycin (10 and 50 μg/mL in the medium, respectively). The culture was identified using molecular analysis of the ribosomal internal transcribed spacer (ITS). To achieve this, the Invitrogen PureLink Genomic DNA kit (Thermo Fisher Scientific, Wilmington, DE, United States) was used for DNA extraction in accordance with the manufacturer’s protocol. DNA was quantified using a NanoDrop 2000 instrument (Thermo Fisher Scientific, Wilmington, DE, United States) at 260 nm. A primer for the ITS gene was selected from the published studies (Mai and Coleman, 1997; Van Hannen et al., 2002) and was used to identify the microalgae (NS7m-F 5′-GGC​AAT​AAC​AGG​TCT​GT-3′ and LR1850-R 5′-CCT​CAC​GGT​ACT​TGT​TC-3′). Amplicons obtained with these primers were purified using a QIAquick PCR Purification kit (Qiagen, Hilden, Germany) and sequenced using Sanger sequencing and capillary electrophoresis (Applied Biosystems SeqStudio, Thermo Fisher Scientific, Wilmington, DE, United States). Both strands were read to ensure the reliability of the sequencing. Sequencing data were assembled using the CAP3 Sequence Assembly Program (Huang and Madan, 1999), and then sequences were manually curated using the eBioX program (version 1.5.1) and analyzed using the BLASTN tool with the nucleotide database (Chen et al., 2015). Finally, to construct the phylogenetic tree, sequences reported by Zou et al. (2016) were used. The MEGA X package was employed using the neighbor-joining method with 1,000 bootstrap values (Sohpal et al., 2010).

### 2.2 Biomass and lipid productivity as a function of nitrate and phosphate concentration

In order to compare the biomass and lipid content in *C. sorokiniana* culture with the nitrogen and phosphorus content, three levels of KNO_3_ (0.19; 0.67; 1.5 g/L) and five levels of KH_2_PO_4_ (0.33; 0.43; 0.82; 1.2; 1.6 g/L) were evaluated while maintaining other nutrients at a constant level and using NaHCO_3_ (1 g/L) as a carbon source in the modified Chu 13 medium (Yeesang and Cheirsilp, 2011) comprising FeCl_3_·6H_2_O 0.0073 g L^−1^ in EDTA 0.00916 g L^−1^; K_2_HPO_4_ 0.04 g·L^−1^; MgSO4·7H_2_O 0.05 g L^−1^; CaCl_2_·2H_2_O 0.04 g L^−1^; KNO_3_ 0.1 g L^−1^; H_3_BO_3_ 0.002859 mg L^−1^; Na_2_MoO_4_·2H_2_O 0.05 mg L^−1^; ZnSO_4_ 0.1234 mg L^−1^; CoCl_2_·2H_2_O 0.05 mg L^−1^; MnCl_2_ 1.146 mg L^−1^; and CuCl_2_·2H_2_O 0.054 mg L^−1^. To maintain the stock, it was reseeded every 3 months (Lugo-De Ossa et al., 2022). Experiments were performed in a 250-mL Erlenmeyer flask containing 120 mL of effective volume and inoculum consisting of 5% (v/v) seed medium taken at the 72nd h. The vessel was maintained under 13 μmol/m^2^-s and a 12:12 photoperiod.

### 2.3 Biomass concentration and lipid content as a function of carbon dioxide and light intensity

The cultures were created in the photobioreactor (PBR) using the modified Chu 13 medium with the concentrations of nitrogen and phosphorus obtained in the previous experiment. The PBR consisted of a vertically mounted glass tubular column. The dimensions of the PBR were 56.0 cm (height) and 34.0 cm (diameter), and the effective volume was 0.2 L, with openings at the top and bottom sides. The thickness of the column wall was 0.34 cm. Mixed gas (air–CO_2_), with a flow rate of 1 vvm, was fed to the column through a 30-mm diameter gas distributor ring located at the bottom of the column. The initial cell density was 0.1 g/L, and light was provided by 3500 K 9-W cool white fluorescent tubes. In all cases, a light/dark cycle of 12:12 h was maintained. The light intensity and CO_2_ concentrations for biomass production and lipid content were optimized using the RSM with a 3^2^ experimental design. The two variables were tested at three levels: light intensity at 13, 39, and 65 μmol/m^2^-s and carbon dioxide at 0.03, 10, and 20%. According to this design, nine treatments for each optimization were employed containing three replications. The relationship between the variables was analyzed using a statistical model by fitting a second-order polynomial equation to the data obtained from the 27 runs (Table 1). All the calculations were made using the data from the last day of the culture (14th day). The response surface analysis was based on multiple linear regressions that considered the main, quadratic, and interactive effects, in accordance with Eq. 1

$$Y=\beta_{0}+\sum_{i=1}^{n}\beta_{i}X_{i}+\sum_{i<j}^{n}\beta_{ij}X_{i}X_{j}+\sum_{j=1}^{n}\beta_{ij}X_{j}^{2},$$ (1)

where Y represents the response of experimental biomass and lipid content; i and j are linear and quadratic coefficients, respectively; β is the regression coefficient; n is the number of variables studied in the experiments; and the Xs are factors (independent variables). The goodness of fit of the model was evaluated by the coefficient of determination (*R* ^2^) and analysis of variance (ANOVA). The significance of regression coefficients was determined with a confidence level of 95%. The statistical analysis and the optimum values for each response variable were found based on mathematical models using Statgraphics Centurion XVI software.

**TABLE 1:** Biomass concentration and lipid content obtained from experimental design with *Chlorella sorokiniana* after 14 days of culture.

Treatment	Light intensity (μmol/m^2^-s)	Carbon dioxide (%)	Biomass g/L (measured)	Lipid content % (measured)
T1	13	0.03	0.44 ± 0.14	37.1 ± 3.5
T2	39	0.03	2.81 ± 0.16	15.8 ± 1.5
T3	65	0.03	3.45 ± 0.20	13.5 ± 4.1
T4	13	10	2.21 ± 0.16	31.4 ± 13.5
T5	39	10	2.02 ± 0.91	15.4 ± 4.4
T6	65	10	2.93 ± 0.30	17.2 ± 1.1
T7	13	20	1.08 ± 0.52	49.1 ± 0.5
T8	39	20	2.28 ± 0.39	22.3 ± 5.1
T9	65	20	1.72 ± 0.29	38.9 ± 1.8

### 2.4 Measurement of variables

#### 2.4.1 Biomass concentration

Dry biomass was measured by absorbance at 437 nm, and the dry weight was calculated according to a calibration curve. The calibration curve was constructed as follows: a representative sample of the culture of known volume was taken and filtered on a pre-weighed filter paper. The filter paper was then left in the muffle furnace at 105°C for 24 h, the filter with the dry cells was weighed, and the biomass weight by difference was calculated (Arredondo and Voltolina, 2007). At the end of each assay, dry biomass was also determined by the described method to confirm the value obtained by the calibration curve.

#### 2.4.2 Nitrate and phosphate concentration

The nitrate concentration was measured using the salicylic acid method reported by Palomino (1997) using a Synergy microplate reader at 410 nm. The calibration curve was constructed from a concentrated solution of KNO_3_. The phosphate concentration was determined by the ascorbic acid method reported by the Standard Methods for the Examination of Water and Wastewater (Health and APHA, 2015) (Baird et al., 2017) using a Synergy microplate reader at 880 nm. The calibration curve was constructed from a concentrated solution of K_2_HPO_4_.

#### 2.4.3 Determination of total lipid content

The lipid content was determined using the gravimetric methodology, by modifying the method of Bligh and Dyer (1959) and breaking the cells by sonication (Bligh EG and Dyer W J, 1959; Hosseini et al., 2018; Lugo-De Ossa et al., 2022). About 15 mL of the culture medium was taken, and the biomass of the culture medium was separated by centrifugation at 15,000 rpm/10 min. The medium was resuspended in 1 mL of chloroform: methanol solution (2:1 ratio) and was subjected to ultrasound for 2 h at 30 Hz. It was later centrifuged at 15,000 rpm/10 min to separate the biomass from the solvent. The solvent phase was recovered with the oils in a previously weighed Eppendorf tube. Extraction was repeated in triplicate, and the solvent layers collected were combined. The Eppendorf tube containing the solvents was placed in an oven for evaporation at 80°C until a constant weight was reached. The mass of the lipid fraction was used to measure the lipid content of the algal cells (g lipids/g dry biomass*100).

#### 2.4.4 Gases and dissolved gases

The carbon dioxide concentration in the water was determined by measuring the alkalinity (Pérez and Restrepo, 2008), and the concentration of dissolved oxygen was determined using a portable oximeter (SI Analytics). Carbon dioxide and oxygen in the gas phase were determined using a multi-gas analyzer (SKY2000-M4).

### 2.5 Development of the mathematical model

This section presents the kinetic growth model of *C. sorokiniana* in a bubble column PBR. The model includes 1) dynamic equations for biomass, phosphate, nitrogen, carbon dioxide, and oxygen; 2) kinetic expressions; and 3) mass transfer models. Dynamic equations are based on mass balances assuming a well-stirred PBR. In this sense, the gas supply has two purposes: first to provide the carbon source for cell growth, and second to provide pneumatic agitation via bubbles that run through the system after having passed through a sprinkler. The kinetic model for microalgae growth was developed using a Monod-type model for multi-nutrient limitations (Eze et al., 2018). Components of the microalgae growth kinetic model include the rate of use of the inorganic carbon, nitrate, and phosphate sources; oxygen production; and pH variations. Mass transfer dynamics are presented for CO_2_ and O_2_. The concentration of bulk aqueous phase oxygen increases due to net oxygen excretion by algae cells and net oxygen mass transfer to the bulk gas phase and decreases due to consumption by microalgal respiration. The concentration of bulk aqueous phase carbon dioxide decreases due to consumption by algal cells during photosynthesis and increases due to excretion by algae cells during respiration and net carbon dioxide mass transfer to the bulk gas phase (Shriwastav et al., 2018). For the model, it is assumed that light intensity and temperature are fixed and there is no water evaporation from the culture. The effect of light intensity on specific cell growth, reduction of light intensity due to biomass concentration, and the material of the bioreactor´s wall were included in the model.

Microalgal biomass is produced through photosynthesis Eq. 2 for autotrophic metabolism (Eriksen et al., 2007; Picardo et al., 2013). The carbon source is carbon dioxide dissolved in the medium, and the energy source is the light supplied by the lamps, in the presence of nutrients, oxygen, carbohydrates, and lipids (as fundamental components of biomass).

$$CO_{2}+0.93\;H_{2}O+0.15{NO}_{3}^{-}+0.002P\to {CH}_{1.71}O_{0.4}N_{0.15}P_{0.002}+1.42\;O_{2}+0.15\;OH^{-}$$ (2)

The kinetic model of cell growth is represented as a function of nitrogen (nitrate), phosphorus (phosphate), and CO_2_ (C) sources and as a function of the incident light (I) falling on the biomass due to photoinhibition (Eq. 3). On the other hand, the light coming from the lamps (*I*_*O*_) is attenuated by biomass (including both absorption and scattering) and by the reactor and its liquid content (Eq. 4). This can lead to light limitation, making it difficult for the light to penetrate into the depths of the culture (Krichen et al., 2021). Material balances by component were carried out for the five most important elements present in the system. This involved obtaining five differential equations that represented the dynamics of biomass (X), nitrate (N), and phosphate (P) as substrates and dissolved CO_2_ (C) and dissolved O_2_ (O_2_) (Eq. 5-9). It is known that the relationship between pH and dissolved CO_2_ is derived from chemical equilibrium theory (James et al., 2013). As the culture grows, a decrease in carbon source concentration will cause an increase in pH in the culture broth. For simplicity, it is reasonable to assume that the increase in pH is proportional to the decrease in carbon dioxide concentrations (Eq. 10) (Zhang et al., 1999; Bekirogullari et al., 2017).

$$\mu =\mu_{\max}\left(\frac{N}{K_{N}+N}\right)\;\left(\frac{P}{K_{P}+P}\right)\;\left(\frac{C}{K_{C}+C}\right)\;\left(\frac{I}{K_{I}+I}\right),$$ (3)

$$I=I_{0}\;\left[1-\alpha \left(1+\frac{X}{K_{IX}+X}\right)\right],$$ (4)

$$\frac{dX}{dt}=\mu X-\mu_{d}X,$$ (5)

$$\frac{dP}{dt}=-Y_{PX}\mu X-\mu_{m_{P}}P,$$ (6)

$$\frac{dN}{dt}=-Y_{NX}\mu X-\mu_{m_{N}}N,$$ (7)

$$\frac{dC}{dt}=-Y_{CX}\mu X+k_{la}^{c}\left({C_{LC}}^{*}-C\right)-\mu_{mC}C,$$ (8)

$$\frac{dO_{2}}{dt}=Y_{O_{2}X}\mu X+k_{la}^{O_{2}}\left({C_{LO_{2}}}^{*}-O_{2}\right)-\mu_{mO_{2}}O_{2},$$ (9)

$$\frac{dpH}{dt}=K\frac{dC}{dt}.$$ (10)

The algebraic differential equations were solved using the MATLAB ode15s routine. In order to determine parameter values for the best fit with the experimental data, the MATLAB fmincon routine was used. This algorithm utilizes the simplex search algorithm (Packer et al., 2011). The objective function to be minimized (Eq. 11) was defined as the sum of the square difference between the experimental and predicted values (y_i_) of the six variables in the model during the culture time. Pearson’s correlation coefficient *R* ^2^ was used to evaluate the degree of fit of the model with the experimental data.

$$\sum_{i=1}^{6}\sum_{t=1}^{n}{\left(y_{i}^{\exp}\left(t\right)-y_{i}^{pred}\left(t\right)\right)}^{2}.$$ (11)

## 3 Results

### 3.1 Isolation and identification of the microalgal strain

**FIGURE 1:**
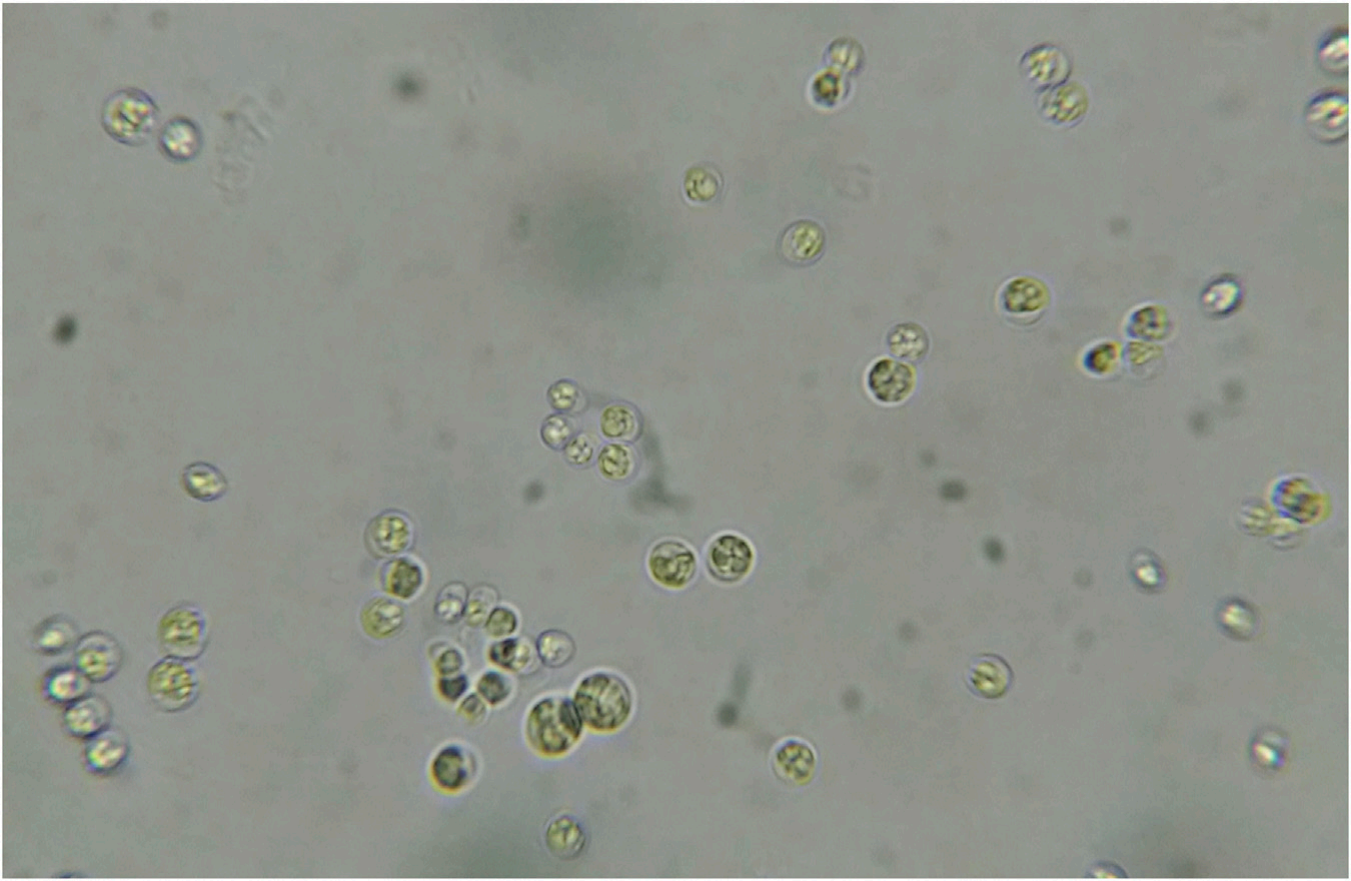
Micrograph of the microalga *Chlorella* sp 1000x.

**FIGURE 2:**
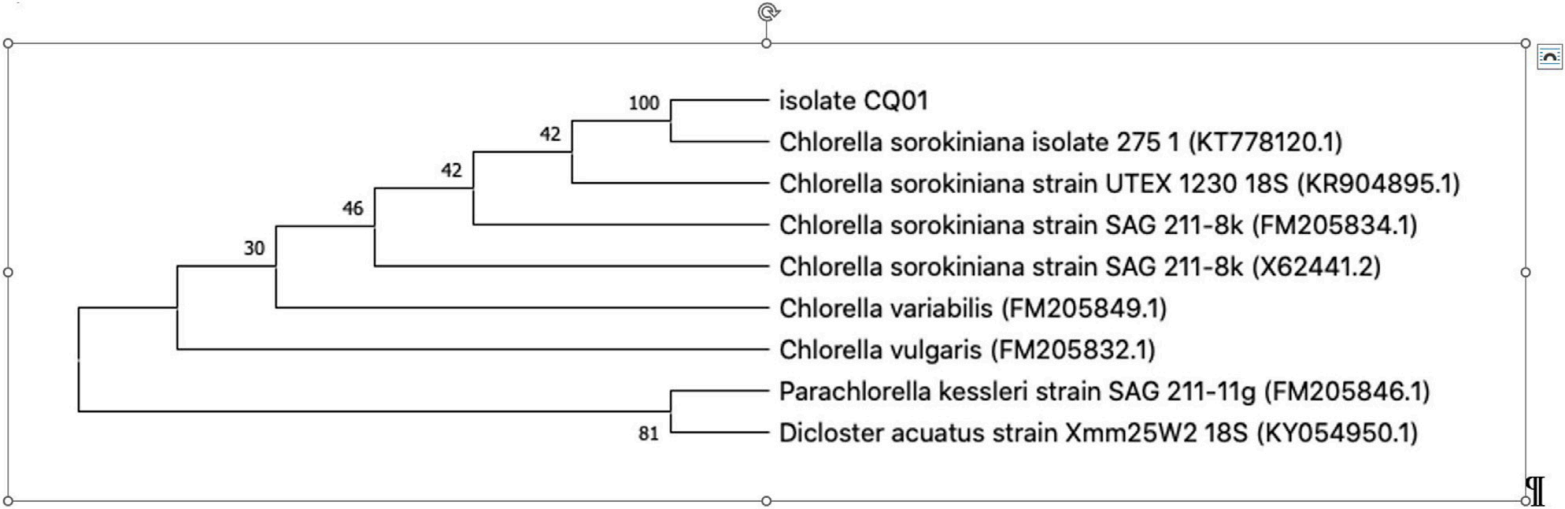
Phylogenetic tree based on 18S rDNA gene sequences.

In this study, morphological characteristics were initially used to identify the isolate CQ01 strain as genus *Chlorella* sp. The cells were solitary, non-motile, and spherical (Figure 1), with a prominent cup-shaped chloroplast. The phylogeny of the CQ-01 strain based on the ITS gene is presented in Figure 2. Based on these data, the strain was identified as *C. sorokiniana*. Analysis was conducted using the MEGAX software (Sohpal et al., 2010) with the neighbor-joining method. *Parachlorella kessleri* and *Dicloster acuatus* were used as outgroups.

### 3.2 Biomass and lipid productivity as a function of nitrate and phosphate concentration

**FIGURE 3:**
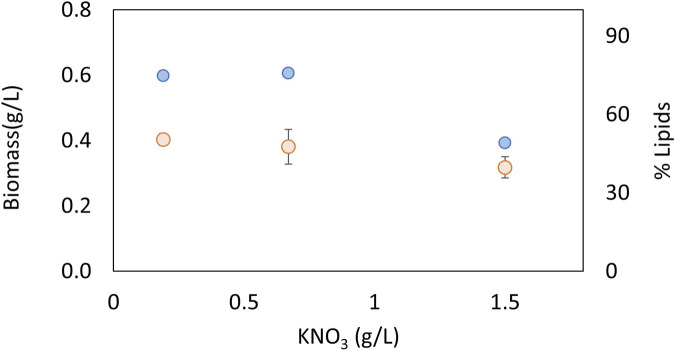
Effect of nitrogen concentration on biomass (blue) and lipids (orange) for an autotrophic culture of *Chlorella sorokiniana*.

**FIGURE 4:**
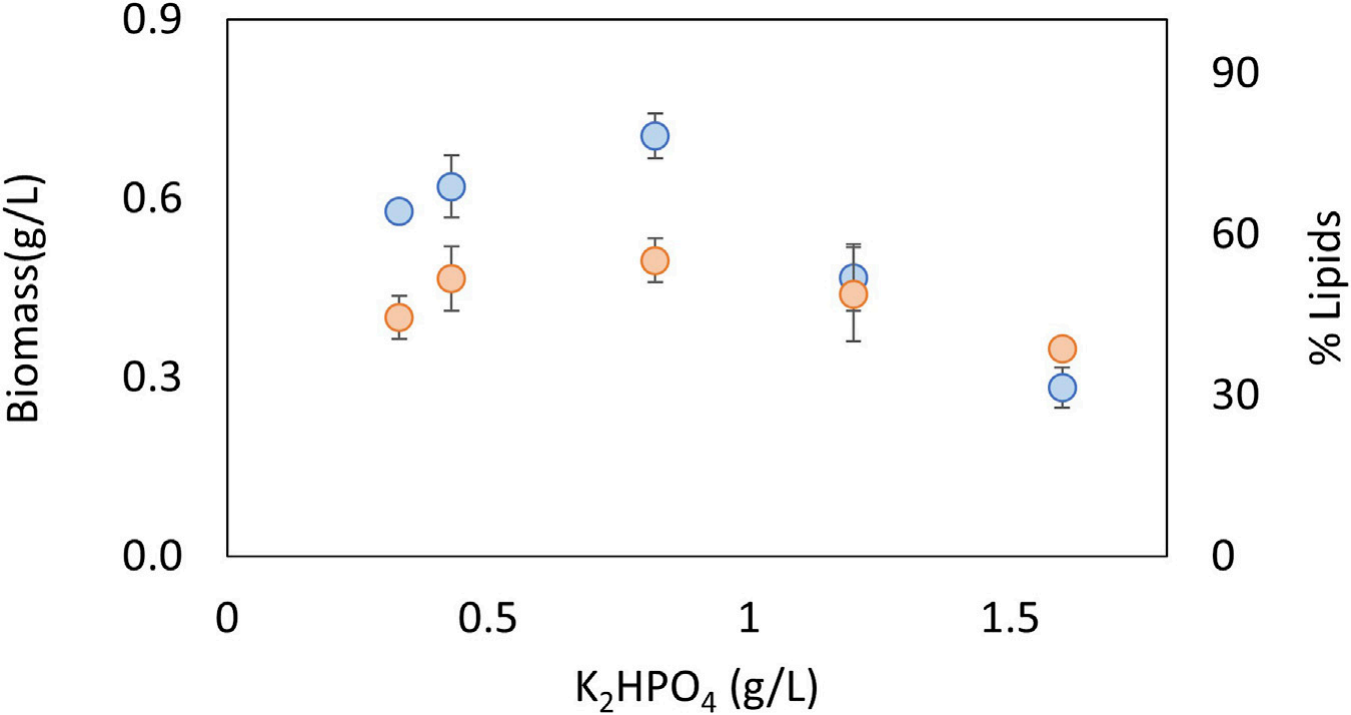
Effect of phosphorus concentration source on biomass (blue) and lipids (orange) for an autotrophic culture of *C. sorokiniana*.

The effects of nitrate and phosphate sources on the production of biomass and lipid content are presented in Figures 3, 4, respectively. ANOVA shows that both factors had a significant effect on biomass concentration. The LSD test evaluated significant differences between the treatments and determined the best concentration to be 0.67 g KNO_3_/L and 0.8 g K_2_HPO_4_/L (*p* < 0.05). In terms of the lipid percentage, the trend clearly shows a higher lipid content with intermediate concentrations of phosphorus and low concentrations of nitrogen. There are no statistically significant differences in lipid content between the treatments (*p* > 0.05). The best concentrations found for biomass were selected for further experiments considering that total lipid productivity increases with an increase in biomass.

### 3.3 Biomass concentration and lipid content as a function of carbon dioxide and light intensity

**FIGURE 5:**
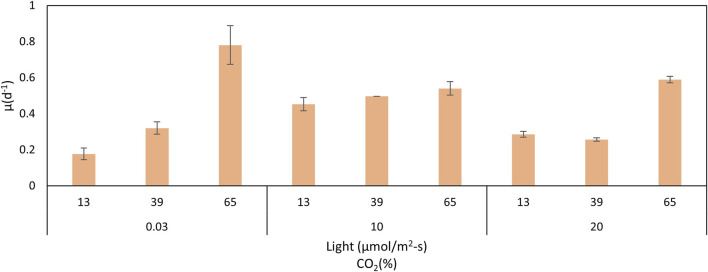
*C. sorokiniana* growth rates under different CO_2_ concentrations and light intensities.

The comparison of biomass and lipid content data is shown in Table 1, and the comparison of growth rates is presented in Figure 5. High growth rates were reached at 10% of CO_2_ and 65 μmol/m^2^-s. However, a clear trend cannot be inferred from these results. The worst growth was obtained at a low CO_2_ concentration (0.03%) and low light intensity (13 μmol/m^2^-s). The effect of the interactions must be considered. For all treatments, there was an adaptation phase from days 0–4, an exponential phase from days 4–8, and a stationary phase, related to the depletion of nitrogen and phosphorus (Almomani, 2020), after 8 days. Biomass production correlates with the cell growth rate (Figure 5). Conditions of high light intensity and low levels of CO_2_ allow higher growth rates.

Growth curves for the lower carbon dioxide concentrations (air) showed that under lower light intensities of 13 and 39 μmol/m^2^-s, *C. sorokiniana* exhibited slow growth and low biomass content. Growth and biomass content increased to 65 μmol/m^2^-s, indicating that the light regime is an important factor controlling biomass in *C. sorokiniana*. As described by other authors (Khoeyi et al., 2012), biomass production in many microalgae increases under high light intensity conditions. Therefore, light intensity causes an increase in reproduction until a saturation point is reached, after which photoinhibition occurs. In this study, photoinhibition was not observed. According to the results, the 65 μmol/m^2^-s light intensity mostly favored the growth rate (3 and 1.5 times greater than 13 and 39 μmol/m^2^-s, respectively). The patterns observed agree with those found by other authors using PBRs, with a similar configuration to this work. In all cases, a specific growth rate was favored at high light intensities (Krzemińska et al., 2015; Metsoviti et al., 2020). However, the fact that photoinhibition was observed at 39 μmol/m^2^-s for high CO_2_ concentrations indicates an interaction between carbon dioxide and light intensity.

Table 1 shows a notable decrease in the total lipid content in the microalgae when irradiance increased for all carbon dioxide concentrations.

Considering the relevance of the interaction between light intensity and carbon dioxide concentration, it is important to analyze the two variables together. This was performed by statistical analysis of the response surface. The response functions in the factor used to predict biomass production and lipid content are given in Eqs 12, 13, respectively.

$$Biomass\;\left(g.L^{-1}\right)=-0.645+0.145A+0.113B-0.0022AB-0.00077B^{2},$$ (12)

$$Lipids\;\left(\%\right)=58.75-1.21A-1.84B+0.0745A^{2}+0.018B^{2},$$ (13)

where the independent variable A is CO_2_ content (%) and B is light intensity (μmol/m^2^-s). A and B are the main effort linear terms while AB is the interaction term, and *A* ^2^ and *B* ^2^ are the quadratic terms involved in the process. The statistical significance of the model equation was analyzed by the F-test for analysis of variance (ANOVA) to the fitted model (Tables 2, 3). The determination coefficient (*R* ^2^) was 0.776 for biomass production and 0.838 for lipid content. This indicated that up to 77.6 and 83.8% of the variations in responses can be explained by the model. The regression of the linear term of light intensity (B) is the most significant factor for biomass production (*p*-value <0.0001), followed by the interaction term (*p*-value of 0.0009) and the quadratic term of light intensity (*p*-value of 0.0311). The quadratic term of CO_2_ does not have a significant effect and was omitted in the model. To understand the effect on lipid content, the regression of linear and quadratic terms of light intensity was the most significant factor, followed by linear and quadratic terms of CO_2_ content. As the interaction term does not have a significant effect, it was omitted from the model.

**TABLE 2:** Kinetic parameters estimated in the microalgae model.

Parameter	Estimated value	Units	Physical meaning
$\mu_{\max}$	2.19	d^−1^	Maximum specific growth rate
$\mu_{d}$	0.0004	d^−1^	Rate of maintenance
$K_{P}$	42	mg/L	Half-saturation constant for phosphate
$K_{N}$	31.5	mg/L	Half-saturation constant for nitrate
$K_{C}$	0.0120	mg/L	Half-saturation constant for carbon dioxide
$K_{I}$	67.34	µmol/m^2.^s	Half-saturation constant for carbon dioxide
$\mu_{m_{P}}$	0.0162	d^−1^	Rate of phosphate uptake for maintenance
$\mu_{m_{N}}$	0.4042	d^−1^	Rate of nitrate uptake for maintenance
$\mu_{mC}$	0.0013	d^−1^	Rate of carbon uptake for maintenance
$\mu_{mO_{2}}$	0.0019	d^−1^	Rate of oxygen uptake for maintenance
$Y_{PX}$	0.116	mg/mg	Phosphate—biomass yield
$Y_{NX}$	0.0315	mg/mg	Nitrate—biomass yield
$Y_{CX}$	0.00095	mg/mg	Carbon dioxide—biomass yield
$Y_{O_{2}X}$	0.0019	mg/mg	Oxygen—biomass yield
$k_{la}^{c}$	3.45	d^−1^	Volumetric gas–liquid mass transfer coefficient for carbon dioxide
${C_{LC}}^{*}$ ^a^	0.81	mg/L	Liquid phase equilibrium concentration of carbon dioxide
$k_{la}^{O_{2}}$	3.87	d^−1^	Volumetric gas–liquid mass transfer coefficient for oxygen
${C_{LO_{2}}}^{*}$ ^a^	6.00	mg/L	Liquid phase equilibrium concentration of oxygen
K_pH_	6.11	L/mg	pH rate constant
α	0.263	(%)	Percentage of the maximum effective light available for the growth
K_ix_	0.0252	mg/L	Half- saturation constant of the biomass concentration

^a^ These parameters were not identified in the optimization.

**TABLE 3:** ANOVA for the response surface quadratic model of biomass concentration.

*Source*	*Sum of squares*	*DF*	*Mean square*	*F-value*	*Prob > F*
A: CO_2_	1.30008	1	1.30008	4.76	0.0419
B: light intensity	10.037	1	10.037	36.73	0.0000
AA	0.863873	1	0.863873	3.16	0.0914
AB	4.20206	1	4.20206	15.38	0.0009
BB	1.48127	1	1.48127	5.42	0.0311
Total error	5.19205	19	0.273266		
Total (corr.)	23.2575	24			

The biomass equation from the response surface adjustment indicates that the coefficients of the linear terms, CO_2_ and light intensity, have a positive effect on increasing biomass concentration. However, the quadratic terms have a negative effect. The lipid percentage equation from the response surface adjustment indicates that the linear terms of CO_2_ and light intensity have a negative effect by decreasing the lipid percentage. However, the quadratic and interaction terms have positive effects.

**FIGURE 6:**
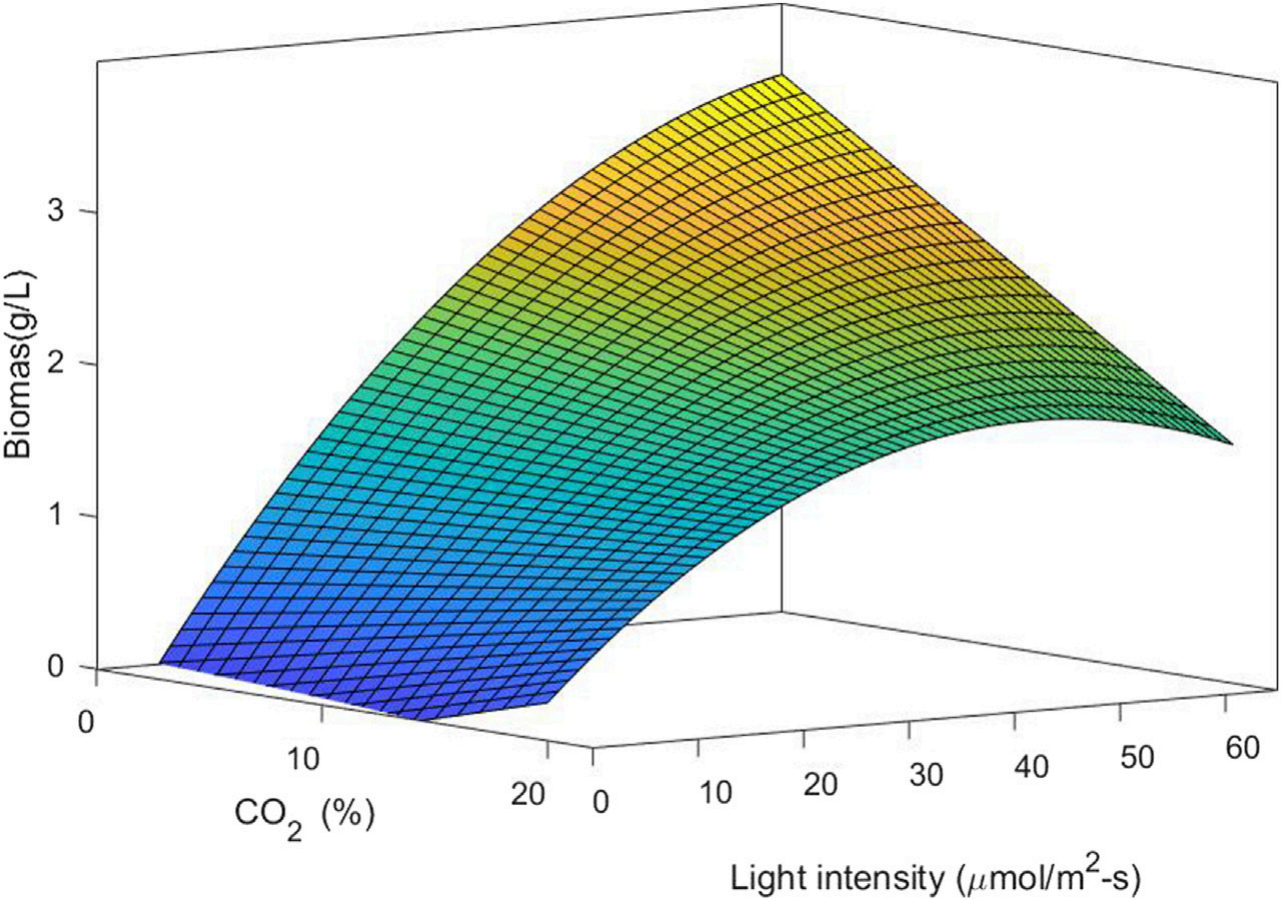
3D surface plots of biomass concentration *vs.* CO_2_ concentration and light intensity.

**FIGURE 7:**
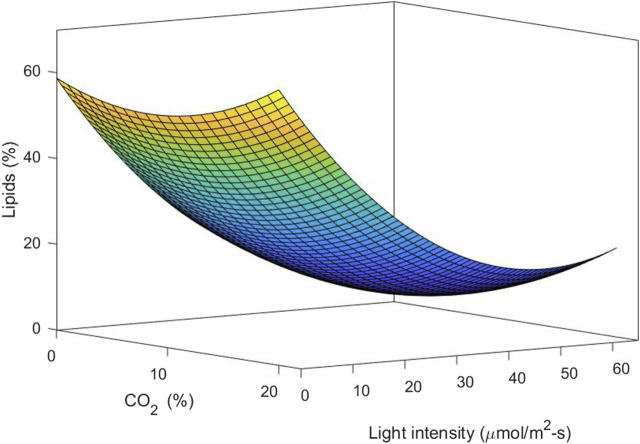
3D surface plots of lipid content *vs.* CO_2_ concentration and light intensity.

Figure 6 shows the response surface and contour plot for biomass concentration. This figure indicates that a low carbon dioxide concentration and the highest light intensity in this study lead to a high biomass concentration. Photoinhibition was not observed for the conditions of light intensity studied. Figure 7 shows the response surface for the lipid percentage. This figure shows that a low light intensity leads to a high lipid percentage, which could be related to lipid accumulation under stress conditions.

#### 3.3.1 Optimization and validation of the statistical model

It is important to simultaneously optimize both lipid percentage and biomass concentration to achieve good lipid productivity. However, each of these response variables is optimized in different light and CO_2_ conditions. The desirability function allows several responses to be optimized simultaneously. In the present study, the optimum value of the desirability function requires a CO_2_ concentration of 20% and a light intensity of 29.9 μmol/m^2^-s for the best lipid and biomass production. The predicted and optimum values for the response variables are shown in Table 4. Experimental validation of the models was performed under the conditions of the model, leading to optimal results. Independent experiments were carried out, and the accuracy of the model was validated using triplicate experiments.

**TABLE 4:** ANOVA for the response surface quadratic model of lipid content.

*Source*	*Sum of squares*	*DF*	*Mean square*	*F-value*	*Prob > F*
A: CO_2_	639.147	1	639.147	19.34	0.0004
B: light intensity	1,102.9	1	1,102.9	33.37	0.0000
AA	262.257	1	262.257	7.93	0.0124
AB	63.9982	1	63.9982	1.94	0.1831
BB	712.514	1	712.514	21.56	0.0003
Error total	528.822	16	33.0514		
Total (corr.)	3,256.24	21			

### 3.4 Mathematical model

**FIGURE 8:**
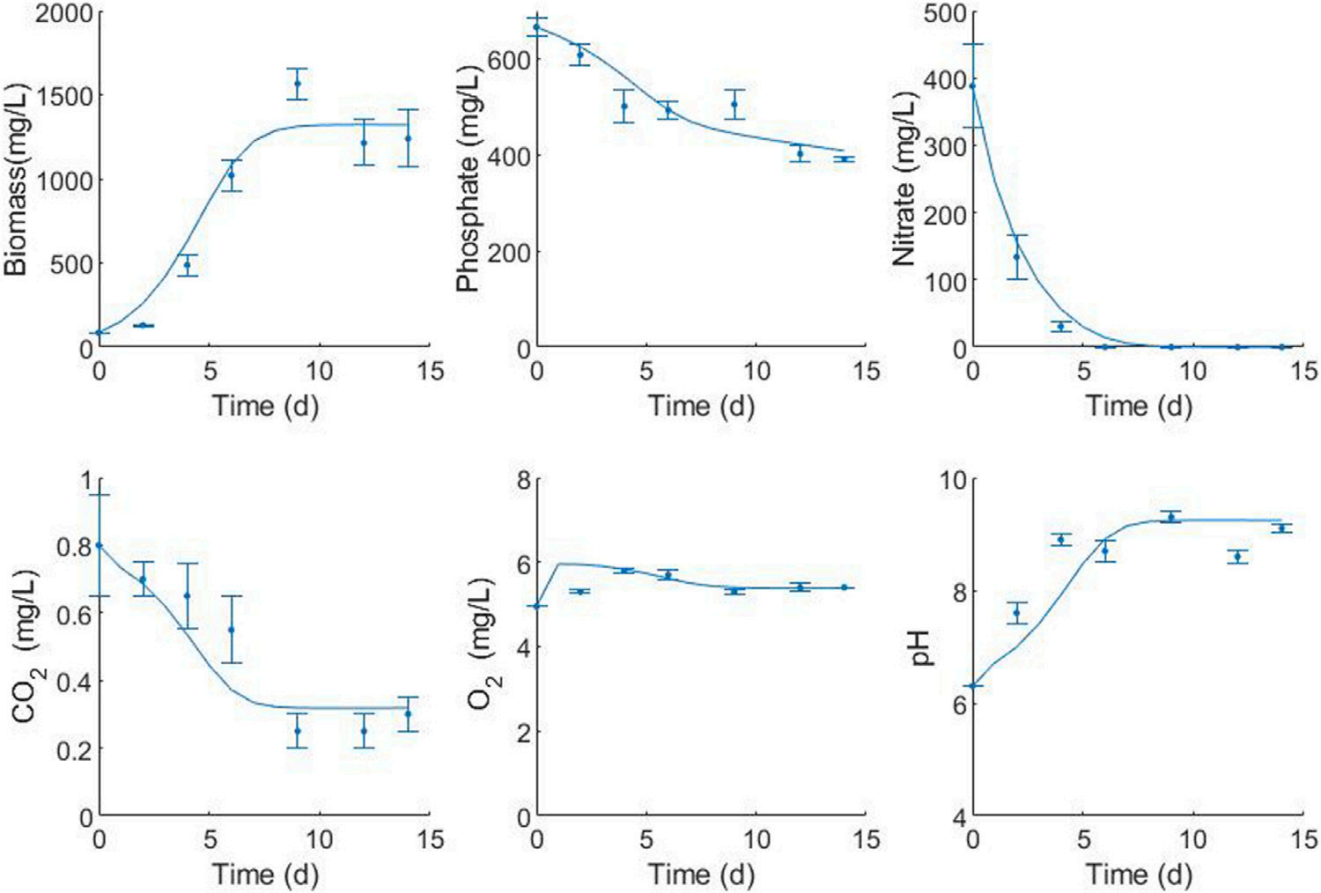
Fitting of the mathematical model for the representative variables of the *C. sorokiniana* culture with the operational factors optimized by the statistical model (29.9 μmol/m^2^-s and 20% CO_2_). Simulation (lines) to experimental data (symbols).

Growth, nutrient uptake, dissolved oxygen, and pH variations for the culture and modeling fits of *C. sorokiniana* under optimized conditions (29.9 μmol/m^2^-s–20% CO_2_ in air) are presented in Figure 8. Nitrogen source consumption in autotrophic conditions was accelerated, indicating that nitrogen is a limiting nutrient for *C. sorokiniana* growth. This source was completely depleted on the 9th day of culture. Likewise, in the case of the P-source, there was a significant decrease in the phosphorus concentration without it being completely consumed within 9 days of culture, indicating an excess of the nutrient. Limited capacity for the removal of total phosphorus in this species has been reported. A very good fit was observed for biomass (0.974), phosphate (0.934), nitrate (0.997), carbon dioxide (0.918), oxygen (0.979), and pH (0.898).

Maximum specific growth rate is one of the most recurrent parameters modeled for microalgae, and a range of 0.077–5.28 days^−1^ is reported in the literature. In the present study, the value of this optimized parameter was 0.61 days^−1^ (Table 5), indicating that a microalgal cell of *C. sorokiniana* requires 1.1 days to duplicate. The rate of maintenance represents the activities carried out by living cells in the absence of growth and is lower than that obtained for *Desmodesmus* sp. (Eze et al., 2018). However, the rates of nitrate uptake for maintenance are very high compared with other maintenance parameters, indicating that nitrogen acts as a limiting substrate. The half-saturation constant or substrate constant is an intrinsic parameter of the cell substrate systems and represents the relative affinity for the substrate. The level of growth-limiting substrates in culture media is normally much greater than half the saturation constant. As a result, growth can be approximated using zero-order kinetics, with the growth rate independent of substrate concentration until it reaches very low values. This can be observed in the case of nitrate concentration after 4 days of culture. Substrate biomass yields are dependent on the stoichiometry of the reaction but are significantly affected by the culture conditions. In the case of the model, the observed yields were used. All yields observed in the present study were very low compared with those in the literature. However, the modeled yields are very similar to the experimental ones for phosphate and carbon dioxide (the Ypx experimental yield is 0.2 mg KH_2_PO_4_/mg biomass, and the Ycx experimental yield is 0.0006 mg/mg biomass). The Y_NX_ experimental yield is 0.3 mg KNO_3_/mg biomass, which is above the predicted value. This could be related to the rate of nitrogen used in the maintenance. According to the model, most of the nitrogen is used for maintenance.

**TABLE 5:** Values optimized by the model and experimental validation results for biomass and lipid production. (Model-optimized factors: Light intensity 29.9 μmol/m^2^-s and CO_2_ 20%.)

	Optimized	Validated	% error
Biomass (g/L)	2.01	1.66 ± 0.09	17
% lipids	30.9	32.8 ± 5.9	6
Lipids (g/L)	0.62	0.54 ± 0.09	13

The mass–transfer coefficient k_L_a is a rate constant that relates to the mass transfer rate, mass transfer area, and concentration change as a driving force. It can be measured or calculated using the published correlations. In the present study, the mass transfer coefficients for oxygen and carbon dioxide were parameters estimated in the model adjustment, and the values were in agreement with those reported by other authors (Eze et al., 2018; Shriwastav et al., 2018). According to the CO_2_ profile, the speed of transfer of CO_2_ is lower than that of its consumption, which limits the mass transfer of this component. It is necessary to increase the Kla to values higher than 3.446 days^−1^ to avoid this limitation. The behavior of dissolved oxygen in the microalgal culture fluctuates according to the photoperiod. In the present study, data on dissolved oxygen were recorded once a day in the light phase. Therefore, where oxygen was produced, the typical oscillation was not observed.

## 4 Discussion

Indigenous strains of microalgae are generally preferred because of their expected stable growth, high adaptability for survival, and productivity. For these reasons, it is crucial to select strains that have a high concentration of proteins and lipids, as they are key components for commercial applications (Duong et al., 2015). The results in the present study show that the isolated microalga belongs to the class Chlorophyceae found in freshwater lakes in Colombia (Aguirre and Bassi, 2013).

Nitrogen and phosphorus are very important nutrients in microalgal growth, meaning that their concentrations need to be optimized for each species and condition. Nucleic acids, proteins, and ATP synthesis require nitrogen. Various coenzymes, key enzymes, and energy substances in the biosynthesis of fatty acids in microalgae are composed of nitrogen (Tongprawhan et al., 2014)**.** Cells first convert nitrate to nitrite using NADH-nitrate reductase and then further metabolize it to ammonia using ferredoxin-nitrite reductase. The ammonia is then converted to glutamate and ultimately to succinate through the action of glutamate decarboxylase. When nitrogen levels are low, lipid production can be promoted as the lack of nitrogen causes the accumulation of carbon precursors in the form of acetyl-CoA, which is a building block for lipid synthesis (Tan and Lee, 2016; Zhu and Huang, 2017). However, nitrogen is required for growth and in high concentrations, such as above 1.5 g/L, can act as an inhibitory substrate (Figure 3). Phosphorus is a crucial macronutrient that plays a role in many metabolic processes, including energy generation, photosynthesis, and signaling pathways. When microalgae are grown under phosphate-limited conditions, they tend to accumulate neutral lipids (Singh et al., 2016). In the present study, higher lipid contents were obtained in intermediate concentrations of phosphate, and also act as an inhibitory substrate at the highest concentrations evaluated of 1.2 g/L (Figure 4). Other authors have found that the optimal concentrations of KNO_3_ and K_2_HPO_4_ to produce lipids in the marine *Chlorella* sp were 0.80 and 0.06 g/L, respectively (Tongprawhan et al., 2014). The optimum nitrogen content was very similar to the value found in the present study, but the phosphate content was very low, indicating how important it is to optimize parameters for each species and strain. Taking into account that the optimum concentrations of C, N, and P are very strain-specific, the C:N:P ratio is widely used to analyze nutrient requirements in microalgae. For example, for *Chlorella vulgaris*, the C:N:P ratio was found to be 2.86:2.71:1 (Almomani, 2020); for the growth of mixed indigenous cultures, the C:N:P ratio was 4.4:1:1.5 (Woertz et al., 2009); and for *C. sorokiniana* under heterotrophic conditions, the C:N:P ratio had little effect on biomass yields and triggered the accumulation of carbohydrates (Lacroux et al., 2021). Taking into account that there is no consensus in terms of a suitable C:N:P ratio for microalgae, it is important to study this ratio for each species and condition. In the present study, the optimum C:N:P ratio was 2.6:1.4:1, which indicates an excess of phosphorus that promotes growth in the native microalgae *C. sorokiniana*.

Light intensity and carbon dioxide concentration are the most studied parameters in microalgal growth and lipid production. In the present study, a decrease in the total lipid content in the microalgae was observed when irradiance increased. Similar results were obtained when *Chlorella protothecoides* microalgae was grown under autotrophic conditions. In this case, there was a decrease in the amount of polyunsaturated fatty acids (PUFAs) and an increase in the intensity of the light used (Krzemińska et al., 2015). For *C. sorokinian*a, it has been reported that in the stationary phase of growth, a decrease in the proportion of these PUFAs was observed (Belkoura et al., 2000). In *Chlorella* sp., the productivity of lipids increased as the light intensity increased, but this trend reversed sharply when the light intensity surpassed 4,500 lux (58.5 μmol/m^2^-s), leading to a decrease in lipid productivity (Tongprawhan et al., 2014). There is currently no consensus on how light intensity affects the lipid content of microalgae. However, it is known that when microalgae are exposed to high intensity of light, the excess light energy absorbed by the photosynthetic apparatus is stored in the form of lipids, specifically triacylglycerol (TAG). The production of TAG requires large amounts of ATP and NAD(P)H, which are generated by photosynthesis. Therefore, lipid accumulation can help dissipate excess light energy and prevent photochemical damage to the algal cells. The impact of light intensity on lipid content is dependent on the ability of individual species of microalgae to capture carbon at high light levels (Binnal and Babu, 2017).

In the present study, the low luminosity studied leads to lipid accumulation, while higher luminosity leads to cellular biomass synthesis. This can be explained by the fact that under stress conditions, many microalgae alter their lipid biosynthetic pathways to produce and accumulate lipids in order to withstand these adverse conditions. Apparently, the increase in light intensity decreases the intracellular accumulation of lipids (low biomass concentrations coincide with high levels of lipid accumulation). This may be due to the fact that the cells are in the active growth phase, which prevents accumulation. When increasing the intensity at different CO_2_ concentrations, the percentage of lipids increases, possibly due to the greater availability of the carbon source.

When performing validation of multiple optimizations through the desirability function, there is a significant error percentage in the biomass obtained (17%). However, in the percentage of lipids, the error is 6%, and in the case of lipid content, the 13% error is due to the fact that the concentration of total lipids depends on biomass. This variability with respect to the statistical model can be attributed to the variability due to the strain. However, it is considered that the statistical model based on the response surface generates an acceptable approximation for the optimization of light and CO_2_ conditions in the native *C. sorokiniana* strain. Multiple optimizations were used to find the conditions that best optimize the percentage of lipids and biomass content, bearing in mind that there is an interaction between the factors. A wider range of these two conditions should be evaluated in order to find the inhibitory light intensity and CO_2_ concentration that best analyze the strain’s behavior.

The desirability function and RSM have been used by other authors in order to optimize culture conditions in microalgae. Supplementary Table S1 presents a compilation of studies on the topic. Differences in optimized conditions indicate that there is no consensus on the best conditions for microalgae growth and lipid production, and for each study, it is necessary to obtain the optimized conditions. However, the RSM is widely applied and useful. Optimized values of CO_2_ concentration in the present study are in agreement with those of other authors (Almomani, 2020), as are the light intensity values (Imamoglu et al., 2014; Tongprawhan et al., 2014; Thawechai et al., 2016) and biomass and lipid content (Thawechai et al., 2016; de Jesus and Maciel Filho, 2017). In the present study, a lipid productivity of 45 mgL^−1^ d^−1^ was obtained, which is in the range reported for other *Chlorella* strains (Tongprawhan et al., 2014).

The best conditions of growth and lipid production in microalgae are spread over a wide range and depend on the species and strains being studied. The best conditions for achieving the highest biomass and lipid content in *Nannochloropsis* sp. were 10% CO_2_ content, 10^7^ cells/mL initial cell concentration, and 0.46 vvm gas flow rate, giving a predicted biomass production of 1.29 g/L and lipid content of 40.3%. With these conditions, the maximum lipid production obtained was 0.52 g/L. The experimental validation of the data was in agreement with the predicted values (Thawechai et al., 2016), which are also within the range of results obtained in the present work. However, feeding a small amount of CO_2_ at a high gas flow rate yielded the maximum biomass content and low lipid content (Thawechai et al., 2016), which was quite different from the trend observed in the present study.

The present study was conducted to characterize a new strain of *C. sorokiniana*. Simultaneous optimization yielded an optimum carbon dioxide concentration of 20%. This could be considered high for a large-scale cultivation system since most industrial exhaust (or flue) gases usually contain between 5 and 15% of carbon dioxide (Lizzul et al., 2014). *C. sorokiniana* has been reported to fix carbon dioxide in a range of 0.03–30%, with an optimum concentration of 15% and fixation rates of 1.56–3.07 g/L (Kong et al., 2021). An optimum concentration of CO_2_ of 15% was also found by other authors, who suggest the use of sequential PBRs as a scaling-up methodology. By using 15-5LPBR, the fixation efficiency was increased to 82.64% (Cam Van et al., 2020). The carbon dioxide concentration was studied for scaling up *C. sorokiniana* cultures in flat panel PBRs, yielding an optimal concentration at a laboratory scale (5 L) of 1.5% with a flow rate of 0.2 vvm. At a pilot scale (18 L), Gabrielyan et al. (2022) found that when keeping the volumetric flow rate of CO_2_ constant, the increase in the rate of aeration noticeably enhanced the growth and biomass yield. They also found that the direct transfer of specific cultivation parameters from a small to a large scale does not ensure a proportional increase in biomass yield (Gabrielyan et al., 2022). To enhance the technology used to capture carbon through microalgae, improvements are required that implement environmentally friendly methods for growing microalgae, such as using flue gas and wastewater treatment technologies. The economic benefits should be maximized by utilizing high-value products obtained from microalgae, and each specific case should be scaled-up and studied (Li et al., 2023).

The effects of carbon dioxide concentration, light intensity, and inoculum size were studied in *Nannochloropsis gaditana* using RMS. Interaction among factors was significant for a fixed inoculum size at a given CO_2_ concentration, and the final dry weight was independent of light intensity. On the other hand, the final dry weight was quite sensitive to CO_2_ concentration, with an optimum value of around 5%. The relative lipid content increased with increasing CO_2_ concentration and was inversely related to inoculum size. The light intensity had virtually no effect on lipid content when analyzing lipid productivity. Biomass had a greater influence than lipid concentration (Hallenbeck et al., 2015). The same conclusions could be drawn from the present study, where variations in lipid content were not as significant as those in biomass concentration**.** For the marine *Chlorella* sp., lipid productivity increased with increasing light intensity until 4,500 lux (58.5 μmol/m^2^-s). Insufficient light intensity can lead to poor utilization of CO_2_ by microalgae and may even result in the consumption of stored lipids. On the other hand, when light intensity exceeds the saturation limit, photoinhibition can occur. This means that the photosystem becomes overloaded, pigments become bleached, and photosystems are destroyed. Therefore, it is important to find the optimal light intensity that provides enough energy for lipid synthesis without causing photoinhibition (Tongprawhan et al., 2014). While photoinhibition of growth was not observed in the present study at 65 μmol/m^2^-s, optimized conditions for lipid production were far below the photoinhibition light intensity.

For *Chlorella vulgaris,* increasing light incidence, CO_2_ concentration, and impeller speed significantly increased cell concentration because there was no photoinhibition or an excess of oxygen in the medium that could cause cell damage. However, increasing CO_2_ concentration and rotational speed did not favor cellular lipid and carbohydrate production. CO_2_ concentration within a medium range was the most important parameter for lipid and carbohydrate accumulation (de Jesus and Maciel Filho, 2017)**.** Numerous researchers suggest that increasing CO_2_ concentration can promote microalgae growth by providing a better carbon source for photosynthesis and inducing the synthesis of relevant compounds. However, in some species of microalgae, an increase in CO_2_ concentration can lead to a decrease in carbohydrate and lipid content or no significant changes in their content (de Jesus and Maciel Filho, 2017)**.** For *C. sorokiniana,* CY1 biomass and lipid production was optimized by designing a lighting system with a submerged LED light and using seawater at a proportion of 50%, a biomass of 2.8 g/L, and a lipid content of 57%. This resulted in a lipid productivity of 153.7 ± 11.3 mg/L/d (Chen and Chang, 2016).

Microalgal growth models describe microbial kinetics as a function of parameters such as fluid dynamics, light radiation, photoperiod, and nutrient concentration. The response variable can be cell or metabolite concentration, photosynthetic activity, or productivity. The specific growth rate as a function of light follows hyperbolic or exponential kinetics, taking into account photoinhibition. For gas–liquid transfer, the CO_2_ balance, carbonate, and the gas–liquid transfer must be considered. One of the approaches employed to model microalgal growth is the use of kinetic equations, such as the logistic, Gompertz, Richards, or Shunute equations. These equations offer a very good adjustment but do not take into account other culture conditions. Therefore, they do not significantly contribute to the understanding of the phenomena; they simply make a description in time. However, the parameters obtained during different experiments can be used as a response variable for statistical analysis or as a comparison to measure the effect of other factors (Aslan and Kapdan, 2006; Çelekli et al., 2009; Montoya-Vallejo and Acosta-cárdenas, 2021). One of the most studied parameters when modeling the growth of microalgae is light intensity. Several authors present models of the specific growth rate as a function of light intensity. They use Monod-type models and exponential and hyperbolic functions with inhibition, among others. The Monod-type model with inhibition is one of the models most widely used by various authors (Camacho Rubio et al., 1999; Concas et al., 2017). Other studies address the effect of light intensity, taking into account that not all incident radiation is used by microalgae, and then define the photosynthetically active radiation (PAR) and photon flux density (PFD) according to the geometry of the system and the composition and concentration of microalgae, among other variables (Yun and Park, 2003; Solimeno et al., 2017; Darvehei et al., 2018). Models in the microalgal culture could also consider temperature, salinity, pH, nutrient concentration, and gas diffusion. The main nutrients modeled in microalgal growth are nitrogen and phosphorus. Monod-type kinetics with inhibition and multiplicative models for several nutrients has been widely used (Çelekli et al., 2009; James et al., 2013; Darvehei et al., 2018). Carbon source and pH are closely related in microalgal growth, and if the pH is kept constant, a nutrient-type equation for carbon is usually used. Furthermore, carbon is considered in excess and is not modeled in most studies (Darvehei et al., 2018). Another important aspect in the models is the behavior of gases, especially oxygen (product of photosynthesis) and carbon dioxide, depending on the type of reactor. Generally, tubular and airlift bioreactors are studied (Acién Fernández et al., 1999; Camacho Rubio et al., 1999; Eze et al., 2018). The model proposed in the present study considers a Monod-type effect of nutrients CO_2_, nitrogen, and phosphorus on the specific growth rate, as well as the effect of light intensity, which considers the increase in opacity of the bioreactor material generated by the adherence of particles to the inner surface and the turbidity caused by the growth of biomass. On the other hand, considering that the experiment carried out under optimal culture conditions was performed where both nitrogen and phosphorus do not present inhibition of cell growth (based on the results presented in Figures 3, 4), this effect was not included in the proposed mathematical model.

Supplementary Table S2 presents the kinetic parameters of the model and compares them to the reference values from the literature. Supplementary Table S2 shows that the values determined from the parameter adjustment algorithm are consistent with those in the literature. While any differences may be related to variations in species and culture conditions, they are in agreement with the experimental values of the cultures studied here. For example, the Yxc value identified with the model was 9.50 × 10^−4^ mg/mg, while the experimental yield was 4.1 × 10^−4^ mg/mg. On the other hand, the saturation constant values for phosphorus, nitrogen, and CO_2_ indicated that nitrogen and phosphorus are the substrates that have the greatest effect on growth, considering that their values are an order of magnitude higher than that for CO_2_, and therefore, limiting concentrations occur at relatively high values. The α value of 0.263 found in the model shows that the effective light intensity significantly reduces by about 75% due to the increase in opacity in the reactor. An increase in light intensity to compensate for this effect may be considered for future experiments.

A growth model for *Chlorella vulgaris* that considers the combined influence of light intensity and total inorganic carbon using a Monod-type multiplicative model was developed by Filali et al. (2011). In this model, the total inorganic carbon consists of carbon dioxide and bicarbonate and carbonate ions. A specific growth rate of 0.08 h^-1^ was obtained, along with a half-saturation constant for a light intensity of 0.14 µE/s10^9^ cells, and a limitation constant for the total inorganic carbon of 1.28e-5 mol/10^9^ cell YX/[TIC] from the stoichiometric equation of biomass (Filali et al., 2011).

A culture of *Chlamydomonas* sp. and *Chlorella* sp. was modeled in a gas-tight PBR. The photosynthetic quotient, taken as the relationship between the oxygen uptake rate and carbon dioxide uptake rate, was used as an independent variable. The model was based on stoichiometric equations, taking into account the nitrogen source and maintaining a constant pH by titration of the gases. The photosynthetic quotient was close to 1 and increased due to oxidative processes when no organic products were secreted from the cells. All CO_2_ taken up was incorporated into the biomass, and the PQ was equal to the oxygen biomass yield (Eriksen et al., 2007). A simple and robust microalgae kinetic model was developed to predict and control *Desmodesmus* sp and *Scenedesmus obliquus* cultivation in wastewater. The effect of nitrogen source and concentration, phosphorus concentration, pH, and carbon source was modeled using a Monod-type model limited by several substrates. Under autotrophic metabolism, the pH increases due to the removal of bicarbonate ions; under heterotrophic metabolism, the pH decreases due to the use of organic carbon sources; and under mixotrophic metabolism, the pH remains stable (Eze et al., 2018). In general, pH values can play a significant role in enhancing algae production by increasing the content of algae, while simultaneously decreasing the presence of invading organisms (Chia et al., 2018).

Batch experiments were conducted to examine how the initial concentrations of nitrogen and phosphorus impact the ability of *Chlorella vulgaris* to remove nutrients. Additionally, biokinetic coefficients, such as the reaction rate constant (k), the half-saturation constant (Km), and the yield coefficient (Y), were calculated using the Michaelis–Menten rate expression, with the chlorophyll content serving as a measure of microalgal growth (Aslan and Kapdan, 2006). A comprehensive mechanistic model to simulate 37 state variables in a PBR was developed to describe algal–bacterial growth dynamics (Shriwastav et al., 2018).

In the present work, an axenic culture was used to study a wild isolated strain of microalgae. However, taking into account natural symbiotic relationships that occur in ecosystems, the effect of the co-culture of microalgae with bacteria should be investigated, especially for the remediation of organic matter; for example, *C. sorokiniana* has been used in co-culture with *Pseudomonas* sp, employing palm oil mill effluent in a newly designed PBR, yielded 5.7 g/L of biomass with a 14.4% of lipid content (Cheah et al., 2020)**.**

## 5 Conclusion

The microalgae *C. sorokiniana* was isolated from a freshwater lake and identified using molecular analysis of the ribosomal ITS. Microalgae can efficiently modify their metabolism in response to changes in environmental conditions. Under optimal growth conditions, substantial amounts of biomass were produced but with relatively low lipid contents. In contrast, under stress conditions, the microalgae altered their lipid biosynthetic pathways toward lipid production and accumulation. According to the results, low luminosity leads to lipid accumulation, while the highest luminosity applied in the present study leads to cellular biomass synthesis. Biomass production correlates with cell growth rate. The highest cell growth rate was found for conditions of high light intensity and low levels of CO_2_. Optimal conditions to maximize lipid content and biomass concentration were obtained using 20% CO_2_ and a light intensity of 29.9 μmol/m^2^-s, with a theoretical biomass concentration of 2 g/L and 31% of lipids. Validation tests of these conditions achieved a biomass concentration of 1.66 ± 0.09 g/L and lipid content of 32.8% ± 5.9%. The results show that stress conditions correspond to low luminosity and high CO_2_ concentration. A multi-parametric model was developed in this study to predict the dynamic behavior of six system variables. A total of 18 kinetic and mass transfer parameters were identified, which were important to understand and model the system. It was found that the nitrogen and phosphorus sources have the greatest effect on growth. The proper fit of the model including the effect of light intensity shows the importance of this variable within the system. It is recommended that a light control should be used that increases the light intensity as the opacity in the system increases.

## Data Availability

The raw data supporting the conclusion of this article will be made available by the authors, without undue reservation.
