# Supplementary material for: Biomass and lipid production by the native green microalgae Chlorella sorokiniana in response to nutrients, light intensity, and carbon dioxide: experimental and modeling approach
Source: Front Bioeng Biotechnol. 2023 May 17;11:1149762. doi: 10.3389/fbioe.2023.1149762 (PMC10229873; doi:10.3389/fbioe.2023.1149762)
Supplement: Supplementary file 1 [file Table1.docx]

Supplementary Material

Biomass and lipid production by the native green microalgae *Chlorella sorokiniana* in response to nutrients, light intensity and carbon dioxide: experimental and modeling approach

Carolina Montoya-Vallejo^*^, Juan Carlos Quintero Díaz, Fernando León Guzmán Duque

*** Correspondence:** Corresponding Author: [carolina.montoya1@udea.edu.co](mailto:carolina.montoya1@udea.edu.co)

**Supplementary Table 1. Optimized culture conditions for microalgae growth and lipid production using RSM**

| **Microalgae specie** | **Optimized CO_2_** | **Optimized light** | **Other culture conditions optimized** | **Biomass (g/L)** | **Lipids** | **Ref** |
| --- | --- | --- | --- | --- | --- | --- |
| *Nannochloropsis gaditana* | 5% (for biomass)  9% (for lipids) | 400 µE/m^2^-s | Inoculum 400,000 counts/µL | 15 g/L |  | (Hallenbeck *et al.*, 2015) |
| *Nannochloropsis sp.* | 10% CO_2_ | 60 µE/m^2^-s | 10^7^ cells/mL initial cell concentration, and 0.46 vvm gas flow rate | 1.29 | 40.3% | (Thawechai *et al.*, 2016) |
| *Scenedesmus sp* | 6.4% |  | a 1.17 m deep top-lit gas-lift bioreactor | 0.4g/L | 198.4g/m^2^ | (Hosseini *et al.*, 2018) |
| *C. pyrenoidosa* |  | 130.77 µE/m^2^-s | NaNO_3_ 1.78g/L | 2.956 g/L |  | (Yadavalli, Rao and Rao, 2013) |
| *Chlorella vulgaris* | 3.77% |  | 4.01 mM sodium nitrate | 1.1 g/L | 20% | (Aguirre and Bassi, 2013) |
| *Chlorella* sp |  | 55 µE/m^2^-s | 28 °C  agitation rate of 168 rpm | specific growth rate of 0.21d^-1^ protein 17.92 µg/100 µL |  | (Imamoglu, Demirel and Dalay, 2014) |
| marine *Chlorella* sp |  | 4500 lux | KNO_3_ 0.8g/L K_2_HPO_4_ 0.06 g/L, initial pH of 7.8, initial cell concentration 10^7.5^ cells/mL, gas flow rate of 0.03 L /min |  | 94.7 mg/L- day | (Tongprawhan, Srinuanpan and Cheirsilp, 2014). |
| *Chlorella kessleri* | 28% | 70 µE/m^2^-s | 29 mM phosphate concentration | specific growth rate of 0.309 d^-1^ |  | (Kasiri *et al.*, 2015) |
| *Chlorella sorokiniana* PAZ | 1127 mg/l HCO_3_ |  | pH 7.86 | 0.891g/l |  | (Zawar, Javalkote and Puranik, 2016) |
| *Chlorella protothecoides* | 6.26% | 5310 lux | pH-6.51, Temperature-28.63 °C Photoperiod-15.36 h:8.64 h Aeration rate 2.92 lpm |  | 274.15 mg/(L- day) | (Binnal and Babu, 2017) |
| *Chlorella vulgaris* (CPCC 90) | 1.6% | above 1076 µE/m^2^-s |  | 2.48g/L | 35% | (de Jesus and Maciel Filho, 2017) |
| *C vulgaris* | 20% | 150 ± 3 µE/m^2^-s | T= 25 °C, gas flow 0.5 vvm total inorganic nitrogen (TN) = 19mg-N/L, Total phosphorous = 7 mg-P/L, COD = 20 mg-COD/L, inoculum = 0.52 mg/L | specific growth rate of 1.4d^-1^ |  | (Almomani, 2020) |

# Supplementary Table 2. Kinetic parameters estimated in the microalgal model.

| **[Parameter](https://www.frontiersin.org/guidelines/author-guidelines#supplementary-material)** | **[Estimated value in the present study](https://www.frontiersin.org/guidelines/author-guidelines#supplementary-material)** | **[Physical meaning](https://www.frontiersin.org/guidelines/author-guidelines#supplementary-material)** | **[Reference value](https://www.frontiersin.org/guidelines/author-guidelines#supplementary-material)** | **[Specie](https://www.frontiersin.org/guidelines/author-guidelines#supplementary-material)** | **[Reference](https://www.frontiersin.org/guidelines/author-guidelines#supplementary-material)** |
| --- | --- | --- | --- | --- | --- |
| [$\mu_{\max}$](https://www.frontiersin.org/guidelines/author-guidelines#supplementary-material) | [2.19d](https://www.frontiersin.org/guidelines/author-guidelines#supplementary-material)^[-1](https://www.frontiersin.org/guidelines/author-guidelines#supplementary-material)^ | [Maximum specific growth rate](https://www.frontiersin.org/guidelines/author-guidelines#supplementary-material) | [1.92d](https://www.frontiersin.org/guidelines/author-guidelines#supplementary-material)^[-1](https://www.frontiersin.org/guidelines/author-guidelines#supplementary-material)^ | *[C. vulgaris](https://www.frontiersin.org/guidelines/author-guidelines#supplementary-material)* | [(Filali](https://www.frontiersin.org/guidelines/author-guidelines#supplementary-material) *[et al.](https://www.frontiersin.org/guidelines/author-guidelines#supplementary-material)*[, 2011)](https://www.frontiersin.org/guidelines/author-guidelines#supplementary-material) |
|  |  |  | [0.077-1.1 d](https://www.frontiersin.org/guidelines/author-guidelines#supplementary-material)^[-1](https://www.frontiersin.org/guidelines/author-guidelines#supplementary-material)^ | *[C. vulgaris](https://www.frontiersin.org/guidelines/author-guidelines#supplementary-material)* | [(Almomani, 2020)](https://www.frontiersin.org/guidelines/author-guidelines#supplementary-material) |
|  |  |  | [3.26 d](https://www.frontiersin.org/guidelines/author-guidelines#supplementary-material)^[-1](https://www.frontiersin.org/guidelines/author-guidelines#supplementary-material)^ | *[Pseudochlorococcum](https://www.frontiersin.org/guidelines/author-guidelines#supplementary-material)* [sp](https://www.frontiersin.org/guidelines/author-guidelines#supplementary-material) | [(Packer](https://www.frontiersin.org/guidelines/author-guidelines#supplementary-material) *[et al.](https://www.frontiersin.org/guidelines/author-guidelines#supplementary-material)*[, 2011)](https://www.frontiersin.org/guidelines/author-guidelines#supplementary-material) |
|  |  |  | [1.83d](https://www.frontiersin.org/guidelines/author-guidelines#supplementary-material)^[-1](https://www.frontiersin.org/guidelines/author-guidelines#supplementary-material)^ | *[Isochrysis galbana](https://www.frontiersin.org/guidelines/author-guidelines#supplementary-material)* | [(Mairet](https://www.frontiersin.org/guidelines/author-guidelines#supplementary-material) *[et al.](https://www.frontiersin.org/guidelines/author-guidelines#supplementary-material)*[, 2010)](https://www.frontiersin.org/guidelines/author-guidelines#supplementary-material) |
|  |  |  | [5.28 d](https://www.frontiersin.org/guidelines/author-guidelines#supplementary-material)^[-1](https://www.frontiersin.org/guidelines/author-guidelines#supplementary-material)^ | *[Chlamydomonas](https://www.frontiersin.org/guidelines/author-guidelines#supplementary-material)**[reinhardtii](https://www.frontiersin.org/guidelines/author-guidelines#supplementary-material)* | [(Bekirogullari](https://www.frontiersin.org/guidelines/author-guidelines#supplementary-material) *[et al.](https://www.frontiersin.org/guidelines/author-guidelines#supplementary-material)*[, 2017)](https://www.frontiersin.org/guidelines/author-guidelines#supplementary-material) |
|  |  |  | [1.7 d](https://www.frontiersin.org/guidelines/author-guidelines#supplementary-material)^[-1](https://www.frontiersin.org/guidelines/author-guidelines#supplementary-material)^ | *[Isochrysis](https://www.frontiersin.org/guidelines/author-guidelines#supplementary-material)**[galbana](https://www.frontiersin.org/guidelines/author-guidelines#supplementary-material)* | [(Zhou](https://www.frontiersin.org/guidelines/author-guidelines#supplementary-material) *[et al.](https://www.frontiersin.org/guidelines/author-guidelines#supplementary-material)*[, 2014)](https://www.frontiersin.org/guidelines/author-guidelines#supplementary-material) |
|  |  |  | [0.9 d](https://www.frontiersin.org/guidelines/author-guidelines#supplementary-material)^[-1](https://www.frontiersin.org/guidelines/author-guidelines#supplementary-material)^ |  | [(Shriwastav](https://www.frontiersin.org/guidelines/author-guidelines#supplementary-material) *[et al.](https://www.frontiersin.org/guidelines/author-guidelines#supplementary-material)*[, 2018).](https://www.frontiersin.org/guidelines/author-guidelines#supplementary-material) |
| [$\mu_{d}$](https://www.frontiersin.org/guidelines/author-guidelines#supplementary-material) | [0.0004 d](https://www.frontiersin.org/guidelines/author-guidelines#supplementary-material)^[-1](https://www.frontiersin.org/guidelines/author-guidelines#supplementary-material)^ | [Rate of maintenance](https://www.frontiersin.org/guidelines/author-guidelines#supplementary-material) | [0.0924 d](https://www.frontiersin.org/guidelines/author-guidelines#supplementary-material)^[-1](https://www.frontiersin.org/guidelines/author-guidelines#supplementary-material)^ | *[Desmodesmus](https://www.frontiersin.org/guidelines/author-guidelines#supplementary-material)* [sp.](https://www.frontiersin.org/guidelines/author-guidelines#supplementary-material) | [(Eze](https://www.frontiersin.org/guidelines/author-guidelines#supplementary-material) *[et al.](https://www.frontiersin.org/guidelines/author-guidelines#supplementary-material)*[, 2018)](https://www.frontiersin.org/guidelines/author-guidelines#supplementary-material) |
| [$K_{P}$](https://www.frontiersin.org/guidelines/author-guidelines#supplementary-material) | [42mg/L](https://www.frontiersin.org/guidelines/author-guidelines#supplementary-material) | [Half saturation constant for phosphate](https://www.frontiersin.org/guidelines/author-guidelines#supplementary-material) | [10.5 mg PO](https://www.frontiersin.org/guidelines/author-guidelines#supplementary-material)_[4](https://www.frontiersin.org/guidelines/author-guidelines#supplementary-material)_[/L](https://www.frontiersin.org/guidelines/author-guidelines#supplementary-material) | *[Desmodesmus](https://www.frontiersin.org/guidelines/author-guidelines#supplementary-material)* [sp.](https://www.frontiersin.org/guidelines/author-guidelines#supplementary-material) | [(Eze](https://www.frontiersin.org/guidelines/author-guidelines#supplementary-material) *[et al.](https://www.frontiersin.org/guidelines/author-guidelines#supplementary-material)*[, 2018)](https://www.frontiersin.org/guidelines/author-guidelines#supplementary-material) |
|  |  |  | [10.5 mg PO](https://www.frontiersin.org/guidelines/author-guidelines#supplementary-material)_[4](https://www.frontiersin.org/guidelines/author-guidelines#supplementary-material)_[/L](https://www.frontiersin.org/guidelines/author-guidelines#supplementary-material) | *[C. vulgaris](https://www.frontiersin.org/guidelines/author-guidelines#supplementary-material)* | [(Aslan and Kapdan, 2006)](https://www.frontiersin.org/guidelines/author-guidelines#supplementary-material) |
| [$K_{N}$](https://www.frontiersin.org/guidelines/author-guidelines#supplementary-material) | [31.5mg KNO](https://www.frontiersin.org/guidelines/author-guidelines#supplementary-material)_[3](https://www.frontiersin.org/guidelines/author-guidelines#supplementary-material)_[/L](https://www.frontiersin.org/guidelines/author-guidelines#supplementary-material)  [4.1mgN/L](https://www.frontiersin.org/guidelines/author-guidelines#supplementary-material) | [Half saturation constant for nitrate](https://www.frontiersin.org/guidelines/author-guidelines#supplementary-material) | [31.5 mg NO](https://www.frontiersin.org/guidelines/author-guidelines#supplementary-material)_[3](https://www.frontiersin.org/guidelines/author-guidelines#supplementary-material)_[/L](https://www.frontiersin.org/guidelines/author-guidelines#supplementary-material) | *[Desmodesmus](https://www.frontiersin.org/guidelines/author-guidelines#supplementary-material)* [sp.](https://www.frontiersin.org/guidelines/author-guidelines#supplementary-material) | [(Eze](https://www.frontiersin.org/guidelines/author-guidelines#supplementary-material) *[et al.](https://www.frontiersin.org/guidelines/author-guidelines#supplementary-material)*[, 2018)](https://www.frontiersin.org/guidelines/author-guidelines#supplementary-material) |
|  |  |  | [0.018mgN/L](https://www.frontiersin.org/guidelines/author-guidelines#supplementary-material) | *[Isochrysis galbana](https://www.frontiersin.org/guidelines/author-guidelines#supplementary-material)* | [(Mairet](https://www.frontiersin.org/guidelines/author-guidelines#supplementary-material) *[et al.](https://www.frontiersin.org/guidelines/author-guidelines#supplementary-material)*[, 2010)](https://www.frontiersin.org/guidelines/author-guidelines#supplementary-material) |
|  |  |  | [65mgN/L](https://www.frontiersin.org/guidelines/author-guidelines#supplementary-material) | *[Chlamydomonas](https://www.frontiersin.org/guidelines/author-guidelines#supplementary-material)**[reinhardtii](https://www.frontiersin.org/guidelines/author-guidelines#supplementary-material)* | [(Bekirogullari](https://www.frontiersin.org/guidelines/author-guidelines#supplementary-material) *[et al.](https://www.frontiersin.org/guidelines/author-guidelines#supplementary-material)*[, 2017)](https://www.frontiersin.org/guidelines/author-guidelines#supplementary-material) |
|  |  |  | [0.0012mgN/L](https://www.frontiersin.org/guidelines/author-guidelines#supplementary-material) | *[Isochrysis](https://www.frontiersin.org/guidelines/author-guidelines#supplementary-material)**[galbana](https://www.frontiersin.org/guidelines/author-guidelines#supplementary-material)* | [(Zhou](https://www.frontiersin.org/guidelines/author-guidelines#supplementary-material) *[et al.](https://www.frontiersin.org/guidelines/author-guidelines#supplementary-material)*[, 2014)](https://www.frontiersin.org/guidelines/author-guidelines#supplementary-material) |
|  |  |  | [24.5mgN/L](https://www.frontiersin.org/guidelines/author-guidelines#supplementary-material) | *[C. vulgaris](https://www.frontiersin.org/guidelines/author-guidelines#supplementary-material)* | [(Aslan and Kapdan, 2006).](https://www.frontiersin.org/guidelines/author-guidelines#supplementary-material) |
| [$K_{C}$](https://www.frontiersin.org/guidelines/author-guidelines#supplementary-material) | [0.012mg CO](https://www.frontiersin.org/guidelines/author-guidelines#supplementary-material)_[2](https://www.frontiersin.org/guidelines/author-guidelines#supplementary-material)_[/L](https://www.frontiersin.org/guidelines/author-guidelines#supplementary-material) | [Half saturation constant for carbon dioxide](https://www.frontiersin.org/guidelines/author-guidelines#supplementary-material) | [124.9mg Total inorganic carbon/L](https://www.frontiersin.org/guidelines/author-guidelines#supplementary-material) | *[Desmodesmus](https://www.frontiersin.org/guidelines/author-guidelines#supplementary-material)* [sp.](https://www.frontiersin.org/guidelines/author-guidelines#supplementary-material) | [(Eze](https://www.frontiersin.org/guidelines/author-guidelines#supplementary-material) *[et al.](https://www.frontiersin.org/guidelines/author-guidelines#supplementary-material)*[, 2018)](https://www.frontiersin.org/guidelines/author-guidelines#supplementary-material) |
| [$\mu_{m_{P}}$](https://www.frontiersin.org/guidelines/author-guidelines#supplementary-material) | [0.0162 d](https://www.frontiersin.org/guidelines/author-guidelines#supplementary-material)^[-1](https://www.frontiersin.org/guidelines/author-guidelines#supplementary-material)^ | [Rate of phosphate uptake for maintenance](https://www.frontiersin.org/guidelines/author-guidelines#supplementary-material) | [0.0924 d](https://www.frontiersin.org/guidelines/author-guidelines#supplementary-material)^[-1](https://www.frontiersin.org/guidelines/author-guidelines#supplementary-material)^ | *[Desmodesmus](https://www.frontiersin.org/guidelines/author-guidelines#supplementary-material)* [sp.](https://www.frontiersin.org/guidelines/author-guidelines#supplementary-material) | [(Eze](https://www.frontiersin.org/guidelines/author-guidelines#supplementary-material) *[et al.](https://www.frontiersin.org/guidelines/author-guidelines#supplementary-material)*[, 2018)](https://www.frontiersin.org/guidelines/author-guidelines#supplementary-material) |
| [$\mu_{m_{N}}$](https://www.frontiersin.org/guidelines/author-guidelines#supplementary-material) | [0.4042d](https://www.frontiersin.org/guidelines/author-guidelines#supplementary-material)^[-1](https://www.frontiersin.org/guidelines/author-guidelines#supplementary-material)^ | [Rate of nitrate uptake for maintenance](https://www.frontiersin.org/guidelines/author-guidelines#supplementary-material) | [0.0924 d](https://www.frontiersin.org/guidelines/author-guidelines#supplementary-material)^[-1](https://www.frontiersin.org/guidelines/author-guidelines#supplementary-material)^ | *[Desmodesmus](https://www.frontiersin.org/guidelines/author-guidelines#supplementary-material)* [sp.](https://www.frontiersin.org/guidelines/author-guidelines#supplementary-material) | [(Eze](https://www.frontiersin.org/guidelines/author-guidelines#supplementary-material) *[et al.](https://www.frontiersin.org/guidelines/author-guidelines#supplementary-material)*[, 2018)](https://www.frontiersin.org/guidelines/author-guidelines#supplementary-material) |
| [$\mu_{\mathrm{mC}}$](https://www.frontiersin.org/guidelines/author-guidelines#supplementary-material) | [0.0013 d](https://www.frontiersin.org/guidelines/author-guidelines#supplementary-material)^[-1](https://www.frontiersin.org/guidelines/author-guidelines#supplementary-material)^ | [Rate of carbon uptake for maintenance](https://www.frontiersin.org/guidelines/author-guidelines#supplementary-material) | [0.0924 d](https://www.frontiersin.org/guidelines/author-guidelines#supplementary-material)^[-1](https://www.frontiersin.org/guidelines/author-guidelines#supplementary-material)^ | *[Desmodesmus](https://www.frontiersin.org/guidelines/author-guidelines#supplementary-material)* [sp.](https://www.frontiersin.org/guidelines/author-guidelines#supplementary-material) | [(Eze](https://www.frontiersin.org/guidelines/author-guidelines#supplementary-material) *[et al.](https://www.frontiersin.org/guidelines/author-guidelines#supplementary-material)*[, 2018)](https://www.frontiersin.org/guidelines/author-guidelines#supplementary-material) |
| [$\mu_{mO_{2}}$](https://www.frontiersin.org/guidelines/author-guidelines#supplementary-material) | [0.0019 d](https://www.frontiersin.org/guidelines/author-guidelines#supplementary-material)^[-1](https://www.frontiersin.org/guidelines/author-guidelines#supplementary-material)^ | [Rate of oxygen uptake for maintenance](https://www.frontiersin.org/guidelines/author-guidelines#supplementary-material) |  |  |  |
| [$Y_{\mathrm{PX}}$](https://www.frontiersin.org/guidelines/author-guidelines#supplementary-material) | [0.116 mg KH](https://www.frontiersin.org/guidelines/author-guidelines#supplementary-material)_[2](https://www.frontiersin.org/guidelines/author-guidelines#supplementary-material)_[PO](https://www.frontiersin.org/guidelines/author-guidelines#supplementary-material)_[4](https://www.frontiersin.org/guidelines/author-guidelines#supplementary-material)_[/mg biomass](https://www.frontiersin.org/guidelines/author-guidelines#supplementary-material) | [Phosphate – biomass yield](https://www.frontiersin.org/guidelines/author-guidelines#supplementary-material) | [0.027 mg PO](https://www.frontiersin.org/guidelines/author-guidelines#supplementary-material)_[4](https://www.frontiersin.org/guidelines/author-guidelines#supplementary-material)_[/mg biomass](https://www.frontiersin.org/guidelines/author-guidelines#supplementary-material) | *[Desmodesmus](https://www.frontiersin.org/guidelines/author-guidelines#supplementary-material)* [sp.](https://www.frontiersin.org/guidelines/author-guidelines#supplementary-material) | [(Eze](https://www.frontiersin.org/guidelines/author-guidelines#supplementary-material) *[et al.](https://www.frontiersin.org/guidelines/author-guidelines#supplementary-material)*[, 2018)](https://www.frontiersin.org/guidelines/author-guidelines#supplementary-material) |
| [$Y_{\mathrm{NX}}$](https://www.frontiersin.org/guidelines/author-guidelines#supplementary-material) | [0.0315 mg KNO](https://www.frontiersin.org/guidelines/author-guidelines#supplementary-material)_[3](https://www.frontiersin.org/guidelines/author-guidelines#supplementary-material)_[/mg biomass](https://www.frontiersin.org/guidelines/author-guidelines#supplementary-material)  [0.004 mgN/mg biomass](https://www.frontiersin.org/guidelines/author-guidelines#supplementary-material) | [Nitrate- biomass yield](https://www.frontiersin.org/guidelines/author-guidelines#supplementary-material) | [0.279 mg NO](https://www.frontiersin.org/guidelines/author-guidelines#supplementary-material)_[3](https://www.frontiersin.org/guidelines/author-guidelines#supplementary-material)_[/mg biomass](https://www.frontiersin.org/guidelines/author-guidelines#supplementary-material) | *[Desmodesmus](https://www.frontiersin.org/guidelines/author-guidelines#supplementary-material)* [sp.](https://www.frontiersin.org/guidelines/author-guidelines#supplementary-material) | [(Eze](https://www.frontiersin.org/guidelines/author-guidelines#supplementary-material) *[et al.](https://www.frontiersin.org/guidelines/author-guidelines#supplementary-material)*[, 2018)](https://www.frontiersin.org/guidelines/author-guidelines#supplementary-material) |
|  |  |  | [0.145 mg N/mg biomass](https://www.frontiersin.org/guidelines/author-guidelines#supplementary-material) | *[Chlamydomonas](https://www.frontiersin.org/guidelines/author-guidelines#supplementary-material)**[reinhardtii](https://www.frontiersin.org/guidelines/author-guidelines#supplementary-material)* | [(Bekirogullari](https://www.frontiersin.org/guidelines/author-guidelines#supplementary-material) *[et al.](https://www.frontiersin.org/guidelines/author-guidelines#supplementary-material)*[, 2017)](https://www.frontiersin.org/guidelines/author-guidelines#supplementary-material) |
| [$Y_{\mathrm{CX}}$](https://www.frontiersin.org/guidelines/author-guidelines#supplementary-material) | [0.001 mg/mg biomass](https://www.frontiersin.org/guidelines/author-guidelines#supplementary-material) | [Carbon dioxide- biomass yield](https://www.frontiersin.org/guidelines/author-guidelines#supplementary-material) | [2mg/mg biomass.](https://www.frontiersin.org/guidelines/author-guidelines#supplementary-material) | *[C. vulgaris](https://www.frontiersin.org/guidelines/author-guidelines#supplementary-material)* | [(Filali](https://www.frontiersin.org/guidelines/author-guidelines#supplementary-material) *[et al.](https://www.frontiersin.org/guidelines/author-guidelines#supplementary-material)*[, 2011)](https://www.frontiersin.org/guidelines/author-guidelines#supplementary-material) |
|  |  |  | [1.821 mg/mg biomass.](https://www.frontiersin.org/guidelines/author-guidelines#supplementary-material) | *[Desmodesmus](https://www.frontiersin.org/guidelines/author-guidelines#supplementary-material)* [sp.](https://www.frontiersin.org/guidelines/author-guidelines#supplementary-material) | [(Eze](https://www.frontiersin.org/guidelines/author-guidelines#supplementary-material) *[et al.](https://www.frontiersin.org/guidelines/author-guidelines#supplementary-material)*[, 2018)](https://www.frontiersin.org/guidelines/author-guidelines#supplementary-material) |
| [$Y_{O_{2}X}$](https://www.frontiersin.org/guidelines/author-guidelines#supplementary-material) | [0.0019 mg/mg biomass](https://www.frontiersin.org/guidelines/author-guidelines#supplementary-material) | [Oxygen- biomass yield](https://www.frontiersin.org/guidelines/author-guidelines#supplementary-material) | [1.27mg/mg biomass](https://www.frontiersin.org/guidelines/author-guidelines#supplementary-material) | *[Chlorella](https://www.frontiersin.org/guidelines/author-guidelines#supplementary-material)* [sp](https://www.frontiersin.org/guidelines/author-guidelines#supplementary-material) | [(Eriksen](https://www.frontiersin.org/guidelines/author-guidelines#supplementary-material) *[et al.](https://www.frontiersin.org/guidelines/author-guidelines#supplementary-material)*[, 2007)](https://www.frontiersin.org/guidelines/author-guidelines#supplementary-material) |
| [$k_{\mathrm{la}}^{c}$](https://www.frontiersin.org/guidelines/author-guidelines#supplementary-material) | [3.45 d](https://www.frontiersin.org/guidelines/author-guidelines#supplementary-material)^[-1](https://www.frontiersin.org/guidelines/author-guidelines#supplementary-material)^ | [volumetric gas–liquid mass transfer coefficient for carbon dioxide](https://www.frontiersin.org/guidelines/author-guidelines#supplementary-material) | [32.64d](https://www.frontiersin.org/guidelines/author-guidelines#supplementary-material)^[-1](https://www.frontiersin.org/guidelines/author-guidelines#supplementary-material)^ | *[C. vulgaris](https://www.frontiersin.org/guidelines/author-guidelines#supplementary-material)*  [Bubble column PBR](https://www.frontiersin.org/guidelines/author-guidelines#supplementary-material) | [(Filali](https://www.frontiersin.org/guidelines/author-guidelines#supplementary-material) *[et al.](https://www.frontiersin.org/guidelines/author-guidelines#supplementary-material)*[, 2011)](https://www.frontiersin.org/guidelines/author-guidelines#supplementary-material) |
|  |  |  | [3.36 d](https://www.frontiersin.org/guidelines/author-guidelines#supplementary-material)^[-1](https://www.frontiersin.org/guidelines/author-guidelines#supplementary-material)^ | *[Desmodesmus](https://www.frontiersin.org/guidelines/author-guidelines#supplementary-material)* [sp.](https://www.frontiersin.org/guidelines/author-guidelines#supplementary-material) | [(Eze](https://www.frontiersin.org/guidelines/author-guidelines#supplementary-material) *[et al.](https://www.frontiersin.org/guidelines/author-guidelines#supplementary-material)*[, 2018)](https://www.frontiersin.org/guidelines/author-guidelines#supplementary-material) |
|  |  |  | [3.87 d](https://www.frontiersin.org/guidelines/author-guidelines#supplementary-material)^[-1](https://www.frontiersin.org/guidelines/author-guidelines#supplementary-material)^ | *[-](https://www.frontiersin.org/guidelines/author-guidelines#supplementary-material)* | [(Shriwastav](https://www.frontiersin.org/guidelines/author-guidelines#supplementary-material) *[et al.](https://www.frontiersin.org/guidelines/author-guidelines#supplementary-material)*[, 2018)](https://www.frontiersin.org/guidelines/author-guidelines#supplementary-material) |
| [${C_{\mathrm{LC}}}^{*}$](https://www.frontiersin.org/guidelines/author-guidelines#supplementary-material) | [0.8mg/L](https://www.frontiersin.org/guidelines/author-guidelines#supplementary-material) | [liquid phase equilibrium concentration of carbon dioxide](https://www.frontiersin.org/guidelines/author-guidelines#supplementary-material) | [4.8 mg/L for air, 321 mg/L for 20% CO](https://www.frontiersin.org/guidelines/author-guidelines#supplementary-material)_[2](https://www.frontiersin.org/guidelines/author-guidelines#supplementary-material)_ |  | [(Shriwastav](https://www.frontiersin.org/guidelines/author-guidelines#supplementary-material) *[et al.](https://www.frontiersin.org/guidelines/author-guidelines#supplementary-material)*[, 2018) equations and Henry’s constants](https://www.frontiersin.org/guidelines/author-guidelines#supplementary-material) |
| [$k_{\mathrm{la}}^{O_{2}}$](https://www.frontiersin.org/guidelines/author-guidelines#supplementary-material) | [3.87 d](https://www.frontiersin.org/guidelines/author-guidelines#supplementary-material)^[-1](https://www.frontiersin.org/guidelines/author-guidelines#supplementary-material)^ | [volumetric gas–liquid mass transfer coefficient for oxygen](https://www.frontiersin.org/guidelines/author-guidelines#supplementary-material) | [4.34 d](https://www.frontiersin.org/guidelines/author-guidelines#supplementary-material)^[-1](https://www.frontiersin.org/guidelines/author-guidelines#supplementary-material)^ |  | [(Shriwastav](https://www.frontiersin.org/guidelines/author-guidelines#supplementary-material) *[et al.](https://www.frontiersin.org/guidelines/author-guidelines#supplementary-material)*[, 2018)](https://www.frontiersin.org/guidelines/author-guidelines#supplementary-material) |
| [${C_{LO_{2}}}^{*}$](https://www.frontiersin.org/guidelines/author-guidelines#supplementary-material) | [6.0 mg/L](https://www.frontiersin.org/guidelines/author-guidelines#supplementary-material) | [liquid phase equilibrium concentration of oxygen](https://www.frontiersin.org/guidelines/author-guidelines#supplementary-material) | [8.7 mg/L for air, 6.6 mg/L for 20% CO](https://www.frontiersin.org/guidelines/author-guidelines#supplementary-material)_[2](https://www.frontiersin.org/guidelines/author-guidelines#supplementary-material)_ |  | [(Shriwastav](https://www.frontiersin.org/guidelines/author-guidelines#supplementary-material) *[et al.](https://www.frontiersin.org/guidelines/author-guidelines#supplementary-material)*[, 2018) equations and Henry’s constants](https://www.frontiersin.org/guidelines/author-guidelines#supplementary-material) |
| [KpH](https://www.frontiersin.org/guidelines/author-guidelines#supplementary-material) | [6.11 L/mg CO](https://www.frontiersin.org/guidelines/author-guidelines#supplementary-material)_[2](https://www.frontiersin.org/guidelines/author-guidelines#supplementary-material)_ | [pH rate constant](https://www.frontiersin.org/guidelines/author-guidelines#supplementary-material) | [0.879 L/g Acetate](https://www.frontiersin.org/guidelines/author-guidelines#supplementary-material) | *[Chlamydomonas](https://www.frontiersin.org/guidelines/author-guidelines#supplementary-material)**[reinhardtii](https://www.frontiersin.org/guidelines/author-guidelines#supplementary-material)* | [(Bekirogullari](https://www.frontiersin.org/guidelines/author-guidelines#supplementary-material) *[et al.](https://www.frontiersin.org/guidelines/author-guidelines#supplementary-material)*[, 2017)](https://www.frontiersin.org/guidelines/author-guidelines#supplementary-material) |
